# Transcriptomic profiling of microglia reveals signatures of cell activation and immune response, during experimental cerebral malaria

**DOI:** 10.1038/srep39258

**Published:** 2016-12-19

**Authors:** Barbara Capuccini, Jingwen Lin, Carlos Talavera-López, Shahid M. Khan, Jan Sodenkamp, Roberta Spaccapelo, Jean Langhorne

**Affiliations:** 1 The Francis Crick Institute, London NW1 1AT, UK; 2 Leiden University Medical Center, Albinusdreef 2, 2333 ZA, Leiden, Netherlands; 3 Department of Experimental Medicine, University of Perugia, 06132 Perugia, Italy

## Abstract

Cerebral malaria is a pathology involving inflammation in the brain. There are many immune cell types activated during this process, but there is little information on the response of microglia, in this severe complication. We examined microglia by genome wide transcriptomic analysis in a model of experimental cerebral malaria (ECM), in which C57BL/6 mice are infected with *Plasmodium berghei ANKA.* Thousands of transcripts were differentially expressed in microglia at two different time points during infection. Proliferation of microglia was a dominant feature before the onset of ECM, and supporting this, we observed an increase in numbers of these cells in the brain. When cerebral malaria symptoms were manifest, genes involved in immune responses and chemokine production were upregulated, which were possibly driven by Type I Interferon. Consistent with this hypothesis, *in vitro* culture of a microglial cell line with Interferon-β, but not infected red blood cells, resulted in production of several of the chemokines shown to be upregulated in the gene expression analysis. It appears that these responses are associated with ECM, as microglia from mice infected with a mutant *P. berghei* parasite (ΔDPAP3), which does not cause ECM, did not show the same level of activation or proliferation.

Malaria is a life-threatening disease caused by *Plasmodium* parasites that are transmitted to people by bites of infected female mosquitoes of the genus *Anopheles*. The primary victims are children under five years old in sub-Saharan African, who present severe syndromes, including severe anaemia and cerebral malaria (CM)^1^,^2^. The underlying mechanisms leading to CM are still not completely understood. Two main hypotheses have been put forward to explain its pathogenesis; the “microvessel obstruction hypothesis”, in which there is an impaired tissue perfusion due to mechanical obstruction of brain microvessels^3^, and the “hyper-inflammatory response hypothesis” proposing a hyper-activation of host immune cells leading to the excessive release of pro-inflammatory molecules in the brain^4^. Several observations however, suggest that both of these processes may be involved in CM pathology^5^.

Microglia, the resident macrophages of the brain, are myeloid cells that are uniquely adapted to the central nervous system (CNS), and which derive from myeloid progenitors coming from the yolk sac during embryogenesis^6^,^7^. In a physiologically normal brain, microglia present a complex morphology with long processed branches, which are used to scan brain parenchyma and detect alterations^8^. When a challenge occurs in the CNS they rapidly migrate toward the site to prevent the spread of the lesion^9^. Activated microglia can subsequently produce inflammatory mediators, including proinflammatory cytokines, chemokines, free radicals and complement which in turn can induce upregulation of adhesion molecules, recruit immune cells and activate other glial cells^10^.

There is little direct information on whether microglial responses contribute to the inflammatory response during CM, but given their role in many CNS pathologies such as Alzheimer’s disease, multiple sclerosis and neuropathic pain^9^, there is a need to understand and describe the effect of *Plasmodium* infection on microglia, and whether they influence the pathology during ECM.

As it is extremely difficult to obtain microglia samples from human CM the majority of pathogenesis studies of the brain have been conducted in animal models, particularly mouse models involving C57BL/6 or CBA mice infected with *Plasmodium berghei* ANKA (PbA)^11^. Although differences between human and mouse pathology require cautious interpretation, observations in mouse models of experimental CM (ECM) show glial cell activation in the brain^12^,^13^. There are not many studies analysing microglia during ECM, one of them has shown that depletion of cells expressing the chemokine receptor, CX3CR1, which includes microglia, during a PbA infection suggests that they may not play a decisive role in ECM, although they can interact with T cells^14^.

Transcriptomic analysis of microglia has been highly successful in delineating molecular patterns implicated in regulating several pathologies^15^,^16^. Therefore we carried out this study to determine whether the transcriptional profile of microglia was altered during *PbA* infection in C57BL/6 as a first step to delineating whether these cells may be involved in the pathogenesis of ECM.

Gene expression profiles from entire brains of mice showing ECM phenotype have been already reported^17^,^18^, but profiles and identification of mechanisms involving single populations of cells in the brain during ECM have not yet been identified. Analysing the whole brain may indicate changes in gene expression of dominant cell types, but alterations in very small cell populations such as microglia may be obscured.

Here we have compared the gene expression profile of microglia isolated from uninfected mice and from mice infected with *PbA* at different time points after infection using Illumina Beadarrays. The cRNA analysis shows that thousands of genes are differentially expressed at two different time points following infection. Analysis of these data identified cell proliferation and immune response activation involving type I IFN signalling in microglia as the most important features. Microglia from the brains of mice infected with a mutant of *P. berghei* lacking dipeptidyl peptidase 3 (DPAP3), Δ*dpap3*, which does not cause ECM, proliferated less and showed lower activation suggesting that the response was associated with ECM. The production of proinflammatory chemokines produced after *in vitro* stimulation of microglia with IFNβ was consistent with a role for Type I IFNs in the activation of microglia in ECM.

## Results

### Global profile of differentially expressed genes in microglia in ECM

C57BL/6 mice were injected with 10^5^ PbA infected red blood cells (iRBC) intraperitoneally, and mortality, parasitemia and clinical scores, indicative of ECM were monitored daily. Mice showed the first signs of ECM around 5 days post-infection (d5) and reached the humane endpoint between d6 and d8 when parasitemia is around 15–20%. A blue staining after perfusion with Evans Blue indicated that the integrity of the BBB has been compromised^19^ (Fig. S1).

**Figure 1:**
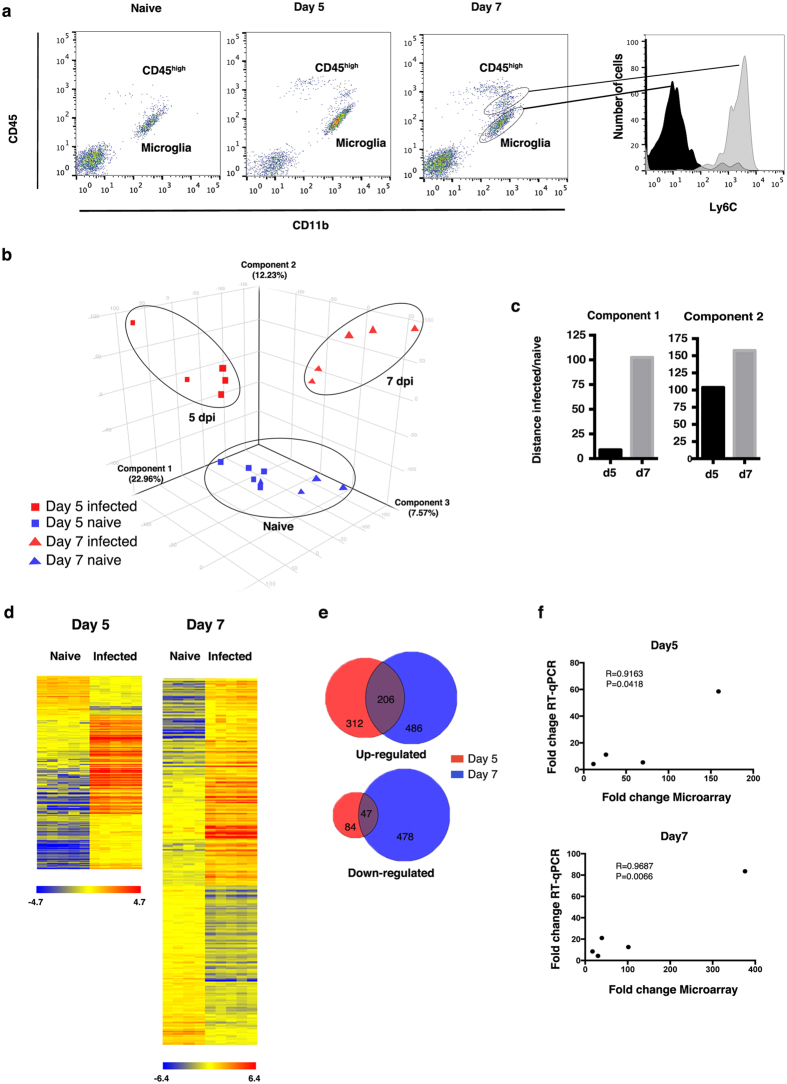
Microglia are activated during ECM. (**a**) FACS plots of microglia, CD11b^+^ CD45^+low^ cells, showing the gates used for sorting, the histogram (right graph) shows Ly6C staining on the isolated microglia (black histogram) compared to brain-infiltrated immune cells (CD45^high^) (grey histogram) (**b**) 3-component representation of Principal Component Analysis showing the 3 different populations according to the infection status: naïve, day 5 post infection and day 7 post infection; (**c**) Graphical representation of the two more significant components (1 and 2) of the PCA showing the distance between infected and uninfected mice for each component. (**d**) Hierarchical clustering of differentially expressed genes in microglia at d5 and d7 post-infection compared to their uninfected controls. 649 and 1217 genes were differentially expressed respectively at d5 and d7, and independently clustered. Each row represents a gene and each column represents an individual mouse. Scale bars underneath represents the normalised intensity of expression of regulated transcripts. (**e**) Venn diagram overlapping differentially expressed genes at day 5 and day 7 post-infection. (**f**) Correlation between mRNA quantification by microarray and RT-qPCR for the differential expression of *Oasl1, IRF7, H2Q8, Cxcl10* and *Ccl5 at d* 5 and at d7. *Ccl5* was less then 2 fold change at d5 in either RT-qPCR or microarray, and it is not reported in the graph. R is the correlation coefficient and P is the *p* value, both have been calculated using Pearson correlation.

Microglia were separated from other brain cells, and brain-infiltrating immune cells (CD45^high^) as CD11b^+^ and CD45^low^ cells^20^. The absence of Ly6C, which is expressed on proinflammatory monocytes^21^, on the sorted microglia populations confirmed their purity (Fig. 1a).

Global gene expression analysis was performed using RNA isolated from sorted microglia on d5, when the mice showed the first symptoms of ECM, and on d7 when the mice reached the humane endpoint. Principal component analysis (PCA) on all statistical unfiltered transcripts normalised across the median of all samples showed 3 distinct populations: naïve, d5 and d7 (Fig. 1b). The samples were separated into three components explaining 22.96%, 12.23% and 7.57% variance, respectively. Analysing the first 2 components, the distances between infected samples and their respective naïve controls were greater at d7 than at d5. The differences in component 1 are 8.84 and 102.4 at 5 and 7 dpi respectively, while in component 2 are 103.80 and 157.62 at 5 and 7 dpi respectively (Fig. 1c). Such differences indicate distinctive levels of microglia activation during the development of ECM.

Statistical testing was further performed to compare infected mice and their uninfected control at each time point. At d5 and d7, 649 and 1217 transcripts respectively, were differentially expressed (DE) (p < 0.005 and fold change > 2) (Fig. 1d,e). As many of the differentially expressed genes were different between day 5 and 7, the filtered transcripts were clustered in 2 separate heatmap trees (Fig. 1d). The different pattern of DE transcripts reflects the distance between infected mice at d5 and d7 showed in the PCA. At d5, 518 transcripts were upregulated compared to their respective naïve controls, accounting for 79.8% of DE transcripts, and 131 (20.2%) were down-regulated (Fig. 1d and Table S1). At d7 the pattern was different, with 692 DE (56.9%) transcripts being upregulated, and 525 (43.1%) down-regulated (Fig. 1d and Table S2).

The gene expression profiles of microglia from d5 and d7 were similar in some aspects to each other, but there were also genes uniquely expressed at each time point. There were 253 DE transcripts shared between d5 and d7, the majority of which (206) were up-regulated compared to their respective controls (Fig. 1e). This group of transcripts includes the most robust responders (fold-change, up to 1178) and contains key molecules involved in the IFNs response (such as *Oas, Oasl, Ifi, Mx, Gbp, IRF* and chemokine families) (Table S3).

The 312 transcripts exclusively upregulated at d5 were mainly involved in the activation of cell proliferation, and included many cell division cycle associated genes (*cdca2, cdca3, ccne2*), molecules supporting the sister chromatid cohesion (*mcm10, dlg7*), plus genes involved in the mitosis and the cell cycle checkpoint (*KIf11, clspn, Tpx2, spag5*). The 84 transcripts down regulated at d5 could not be ascribed to a common biological function.

There were 964 transcripts exclusively differentially regulated at d7. Among the 486 up-regulated transcripts were a large number of genes involved in the immune response such as chemokines (*Ccl5, Ccl8*), genes encoding the MHC class I complex (MHC-I) (*H2-Q5, H2-T22, H2-T23, H2-T4*), Interferon-related genes (*IFI204, IFI205, IFIT3*), GTPases (*Irgm, Garnl4, Arhgap24*), and others. The 478 genes down regulated at d7 showed a smaller fold change (up to −92) compared to changes in the up-regulated genes (up to 1739). Within this group, some genes are related to impaired cell proliferation (*pnma2, Thrsp, AEBP2, Pfkp, Angptl7*).

Despite the high yield of transcripts that passed the statistical filtering, there could be a significant rate of false negatives, which may increase the confidence of the analysis. In order to verify that, we clustered the 253 significant DE transcripts shared between d5 and d7 and compared them to 61 non-significant counterparts (Fig. S2). The directional trend of the two clusters was consistent; the majority of the transcripts were more differentially regulated at d7 than d5 with the exception of a small group of genes, which were more upregulated at day 5. As we expected the non-significant transcripts were differentially expressed in the same direction but at a reduced significance.

Five representative DE genes (*Oasl1, IRF7, H2Q8, Cxcl10* and *Ccl5*) were selected from the microarray analysis for validation by RT-qPCR. In an independent experiment, microglia were collected from naïve mice and infected mice at d5 and d7. Results obtained from microarray and RT-qPCR were compared. Correlation coefficients (R) of 0.9163 and 0.9687 respectively at d5 and d7 and statistical significance (p < 0.05) indicated significant correlation between the two techniques (Fig. 1f).

### Gene Ontology analysis and functional enrichment of the transcriptomes from microglia reveals cell replication and response to Type I Interferons during ECM

We performed Gene Ontology (GO) analysis to investigate further the potential biological roles of the differentially expressed genes. At d5 within the GO category “biological processes”, there was significant enrichment in the GO terms “immune response”, and “antigen presentation”, and 40 GO terms were related to “cell cycle”. Within the “cellular components” category another 17 GO terms related to “cell cycle” displayed enrichment as well as two GO terms related to “antigen presentation”. Finally within the “molecular functions” section, 14 GO terms were also related to “cell cycle” (Fig. S3a,b).

At d7 the gene expression pattern showed enrichment in one GO term for “immune response” and three for “antigen presentation” within “biological processes” category, while, only two terms for “antigen presentation” were enriched within “cellular components” category. In contrast to d5, at d7 there was no enrichment of terms for “cell cycle” (Fig. S3a,b). Terms related to “immune response” and “antigen presentation” were significantly enriched at both time points. Genes belonging to these categories included proinflammatory chemokines able to recruit immune cells to the site of an eventual challenge or injury such as *Cxcl10, Ccl8, Ccl5*; guanylate binding proteins *GBP2, GBP4, Gbp6, Tgtp*; MHC-I complex genes such as *H2-Q8, H2-sg17, H2-Q7, H2-Q2, H2-Q5, H2-K1, H2-T23, H2-D1, H2-D4*; Interferons (IFNs) and related genes and, especially, early Type I IFN response genes such as *Oasl1, Oasl2, Ifit3, Irf7*. Pie charts of the enriched GO terms (Fig. S3b) highlight that the most significant enrichment of “cell cycle” was significantly enriched at d5 while at d7 there was almost exclusively enriched for “immune response”.

To investigate which genes were involved in these processes, we designed a two-step approach to map the molecular pathways affected in microglia during ECM. Genes that were differentially expressed in both days were used as input for TopFunn and GSEA using a False Discovery Rate (FDR) threshold of 0.05. The results of these two suites were strongly consistent with the GO terms from the first round of analysis. Based on our microarray data, the most enriched pathways related to the transcriptome at d5 are involved in cell division (Fig. S5). Gene families, such as, cell cycle-associated proteins (*CDCA2, CDCA5, CDCA8, CDC25B*), cyclins (Cdkn3, Cdkn2d, Cdkn1a, Cdkn1a, Cdkn1a, Cdk5rap2), transcription factors (*E2F1, E2F2, EZH2, IRF1, UHRF1*) and inhibitors of apoptosis (*BIRK5, SKP2*) were upregulated, suggesting that cell proliferation was taking place. In contrast, at d7 the main enrichment involved pathways related to the immune response. The most significant among them is Type I IFN (Fig. S6).

There are many genes known to be affected by downstream of IFN signalling, several of which were upregulated in our set of genes such as chemokines (*Ccl5, Ccl12, Cxcl9, Cxcl10*)^22^,^23^, cytokines (*TNFSF13B*)^24^, proteins involved in antigen presentation (*H2-Q8, H2-sg17, H2-Q7, H2-Q2, H2-Q5, H2-K1, H2-T23, H2-D1, H2-D4, H2Q5, H2T22, H2T23, H2T4*)^25^, transcription factors (STAT2, IRF1) and Interferon induced IFIT, IFITM and OAS (IFIT2, IFIT3, IFITM1, IFITM2, IFITM3, Oas1, Oas2, Oals1). Interestingly, Type I IFN receptor (IFNAR) exhibited upregulation in our dataset.

**Figure 2:**
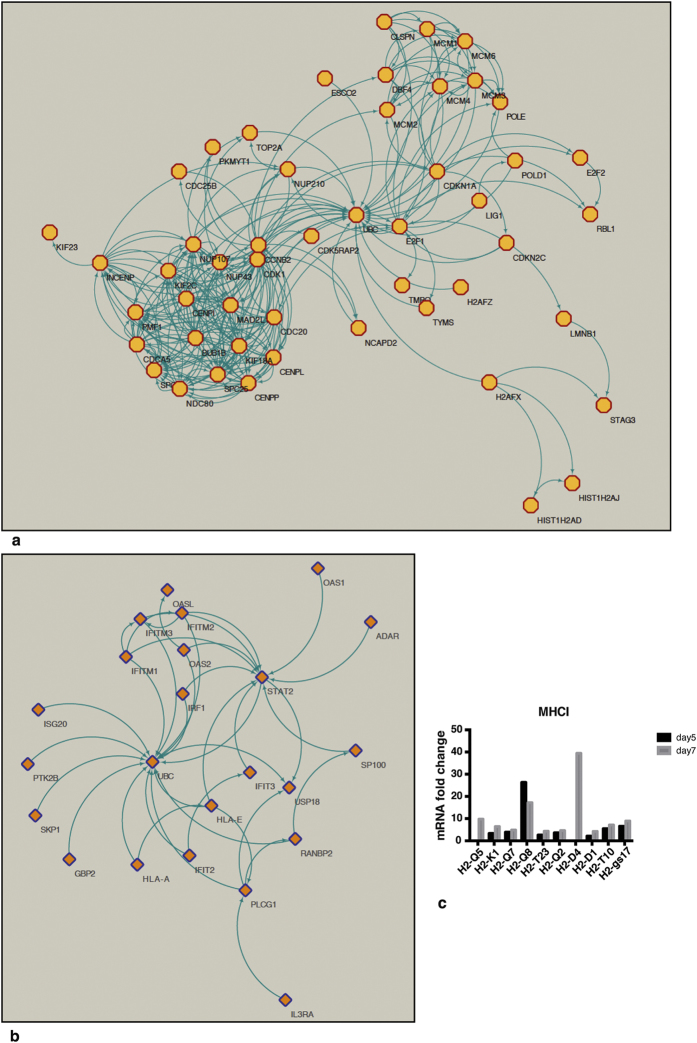
Pathways enrichment reveals activation of cell cycle at d5 and Type I IFN downstream regulation at d7. Pathway mapping were realised using REACTOME and showed by Cytoscape. The network among DE genes at the two time points was showed for “cell proliferation” at d5 (**a**) and “immune response by Type IFN” at d7 (**b**). The blue arrows represent molecular relationship, while the yellow dots represent DE transcripts involved in the networks (**c**) MHC class I DE genes has been displayed as fold change in the infected mice at the two time points compared to their infected control.

Using the REACTOME plugin for Cytoscape, we showed the relationship between DE genes differentially expressed which are involved in cell cycle at d5 (Fig. 2a) and Type I IFN pathway at d7 (Fig. 2b). Because Reactome and ToppFunn use the human nomenclature, some genes have been converted to the human nomenclature prior to the analysis; in particular at d7 genes belonging to MHC class I family are represented as HLA-A and HLA-E. To clarify this discrepancy, we displayed all the mouse MHC class I genes that are differentially expressed and their fold change at d5 and d7 (Fig. 2c).

All together, these results strongly suggest that microglial cells replicate and that they are activated to a proinflammatory phenotype in the acute phase of ECM and that this activation is mainly due to a Type I IFN signal.

### *Ex vivo* analysis of microglia from mice with ECM confirms cell proliferation, production of proinflammatory chemokines, and increased expression of MHC class I molecules

**Figure 3:**
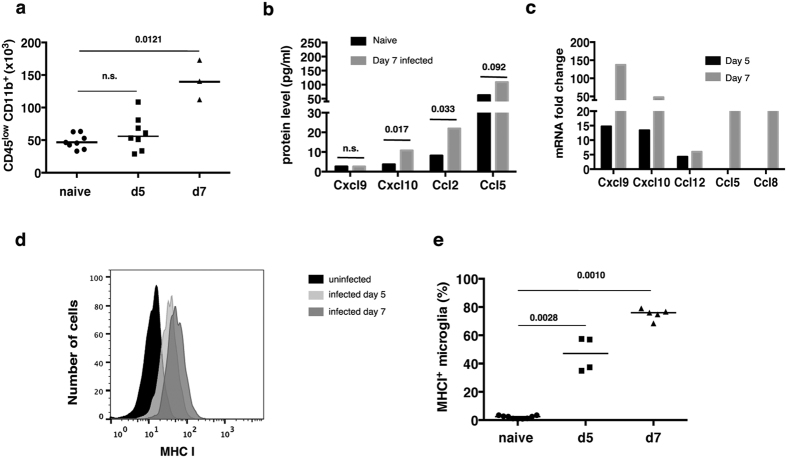
Validation of microarray analysis. (**a**) Number of microglia isolated from naïve mice and infected mice at 5 and 7 days post infection from entire brains. Each dot represents a single mouse and the lines define the median. Statistical analysis was performed by Mann-Whitney test, the number over the bar indicates the *p* value. (**b**) Differentially expressed chemokines in infected mice at day 5 and 7 post infection determined from the microarray analysis. (**c**) Chemokines in the supernatant of microglia was isolated from mice that had reached the humane endpoint 7 days post infection compared with the levels in uninfected mice. Results are expressed as median, n = 7 replicates for 3 separate experiments Statistical analysis was performed using Mann-Whitney test; the number over the bar represents the *p* value. (**d**) Flow cytometry: histograms showing the level of expression of MHC class I molecules on the surface of microglia from naïve mice, and from mice infected with PbA for 5 or 7 days. (**e**) Percentage of MHC class I positive microglia in the brains at day 5 and day 7 post infection of infected mice compared with uninfected naïve mice. Each dot represents a single mouse and the lines define the median. Statistical analysis was performed by Mann-Whitney test, the number over the bar indicates the *p* value, *n.s*. stand for non significant and indicates *p* > *0.05*.

Analysis of the transcriptome suggested that microglia were activated during ECM, and that they proliferated and produced chemokines. In line with these results, we found a significant increase in the number of microglia extracted from brains of mice at d7 compared to uninfected controls (Fig. 3a).

Microglia isolated from d7 also produced the chemokines Ccl2, Ccl5, Cxcl10 on short *in vitro* culture (Fig. 3b), in agreement with the transcriptomic data (Fig. 3c). Although Cxcl9 RNA was increased on the microarray at d7, this chemokine was not present in supernatants from microglial cultures. The discrepancy between Cxcl9 mRNA and protein expression may be due to post-transcriptional regulation of expression of this protein, as shown previously^26^. It was not possible to determine the presence of Ccl8 and Ccl12 proteins, which were significantly up-regulated at the RNA level, because of the absence of specific antibodies in the CBA panel. Interestingly, there were significant amounts of Ccl2 in supernatants, a homologue of Ccl12, which is structurally and functionally similar to both Ccl8 and Ccl12; in fact, these three molecules share the same receptor (CCR2)^27^.

Flow cytometry analysis revealed an increase in the number of MHC-I^+^ microglia obtained from infected mice compared with those of naïve mice, in agreement with the transcriptomic data (Fig. 3d). During the progression of the infection both the number of MHC-I^+^ cells and the Mean Fluorescent Intensity of expression on the surface of the microglia from infected mice were increased (Fig. 3d,e).

### Comparison of the microglia response in ECM with that of a mutant non-ECM *P. berghei*

**Figure 4:**
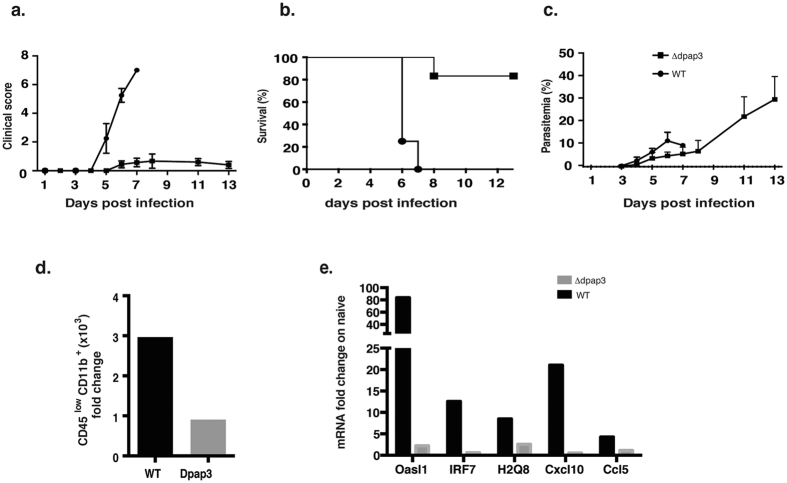
C57BL/6 mice were infected with 105 DPAP3KO iRBC. (**a**) Clinical score of the mice was calculated as the sum of several neurological signs and phenotypic features. Survival (**b**) and parasitemia (**c**) were monitored daily from d3. Results are represented as mean ± SD, n = 5. (**d**) Microglia cell count of sorted cells reveals significant difference in PbA infected mice showing ECM 7 days post infection compared to uninfected mice, on the contrary non-ECM mice infected with ΔDPAP3 have no difference. Statistical analysis was performed by Mann-Whitney test, the number over the column indicates the *p* value. *N.s*. stand for non significant and indicates *p* > *0.05*. (**e**) mRNA fold change of several genes involved in the Type I IFN response revealed by real time PCR. At d7 microglia from non-ECM mice infected with ΔDPAP3 parasites have a lower fold change compared to the ECM mice at the same time point. Results are expressed as mean (n = 5).

In order to determine whether the changes in microglia revealed in the microarray were generated only in an infection that gave rise to ECM, we compared responses of microglia obtained at day 7 from ECM-causing *PbA* infection with that of a mutant of PbA, which does not cause ECM. For this we used a gene-deletion mutant for the dipeptidyl peptidase 3 (DPAP3), Δ*dpap3*^28^. Infection with 10^5^ (Fig. 4a–c) or 10^4^ iRBC (Fig. S4) of PbA Δ*dpap3* resulted in a gradually increasing parasitemia and approximately 85% to 100% survival of mice up to 20 days of infection, with low or no clinical signs of ECM. For the first 5 to 7 days of infection the parasitemias were similar to those of *wt* PbA.

There was no significant increase in the numbers of microglia recovered at day 7 from the PbA Δ*dpap3* infection in contrast to the 3-fold increase observed in microglia from the wt PbA infection (Fig. 4d). We performed quantitative RT-qPCR on microglia isolated from both infections for the genes, *Oasl1, IRF7, H2-Q8, Cxcl10 and Ccl5*. Only microglia from *wt* PbA infections causing ECM show significant fold increases in expression of any of these genes (Fig. 4e). These data suggest that activation of microglia may be feature of ECM.

### IFNβ activates microglia *in vitro*

**Figure 5:**
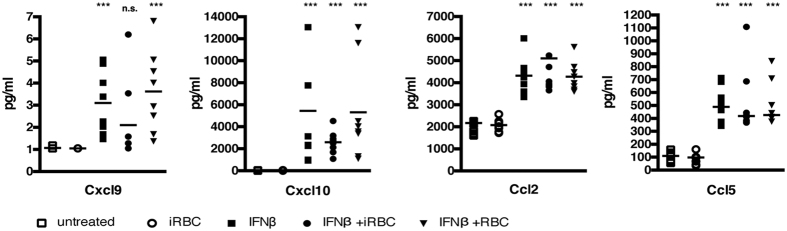
Quantification of proinflammatory chemokines in the supernatant of BV2 cells pre-treated with IFNβ and incubated with iRBC or RBC for 24 h. Results are expressed as median and each dot represent a replicate, n = 8 replicates for 3 separate experiments. Statistical analysis was performed using Mann-Whitney test. ***Represent significance with a *p value* < *0.001*, n.s. stand for non significant and indicates *p* > *0.05.*

Functional enrichment analysis suggested that Type I IFNs might be activating microglia during the acute phase of ECM. To investigate this we stimulated a mouse microglia cell line (BV2^29^), with IFNβ *in vitro* for 24 hours, or with *PbA* iRBC. After 24 hr of culture, the presence of proinflammatory chemokines was determined. Microglia were activated in the presence of IFNβ to produce chemokines, (Fig. 5). Cxcl9, Cxcl10, Ccl2, and Ccl5 could be detected in the supernatants of BV2 cells treated with IFNβ compared to untreated cells, thus reflecting the results of the transcriptome analysis (Fig. 3c) as well as the *ex vivo* experiments using microglia extracted from brains of infected mice undergoing ECM (Fig. 3b). By contrast, incubation of BV2 cells with iRBC did not induce chemokine secretion, nor augment the amount of chemokines produced in response to IFNβ. These results suggest that microglia can be activated by Type I IFNs, but that interaction with infected RBCs is not necessary for their activation.

## Conclusion

Microglial cells are important immune cells that scan brain parenchyma. They are highly plastic and respond to numerous changes in the brain. Here we demonstrate that microglia are activated in an experimental model of cerebral malaria using gene expression microarray. There were clear differences in gene expression profiles of microglia extracted from day 5 and day 7 of a PbA infection in C57BL/6 mice compared with those of microglia from naïve mice, indicating activation of these cells. Microglia from mice infected with a mutant of PbA (ΔDPAP3) that did not cause ECM, were not similarly activated suggesting that the microglial response was a feature of ECM. The transcriptomic profile strongly indicated that the microglial response was driven by Type I IFNs, implying a role for this cytokine in the activation and function of microglia during ECM.

Until now all microarray analyses of brains have shown that a number of genes were over-expressed at the early stage of infection in ECM resistant mice and down-regulated in ECM sensitive strains, indicating an early transcriptional response in resistant mice. While sensitive mice showed upregulation of genes involved in pro-inflammatory networks and Interferon associated response at late stage of ECM^17^,^18^. It was not possible in these whole brain analyses to assign responses to particular cell types. Microglia comprise only a very small proportion of the cells within the brain. Analysing the transcriptome of isolated microglia rather than whole brain allows a deeper insight into how these cells, specifically, respond during ECM, and offers the possibility of predicting which signals may activate them. We used gene ontology, pathway and network analysis to unravel the biological processes involved in activation of microglia during ECM. At 5 days post-infection at the onset of cerebral symptoms, we detected a strong transcriptomic signal for cell-cycle progression, suggesting that there was cell proliferation of microglia in the brain and this was verified by the subsequent observation of increased numbers of cells by 7 days post-infection. Our results in this ECM model are in line with previous studies in other brain-related pathologies showing that microglia are among the first cells to respond to any kind of stimulus in the brain parenchyma^7^, and that they are able to replicate^9^,^30^. In this regard, we provide molecular validation for earlier observations of changes in morphology of microglia in ECM, suggestive of activation^31^, and more recent work using quantitative automated histology showing microglia in a more activated state^32^.

Although there was increased expression of genes involved in activation of immune responses and antigen presentation via MHC-I already at 5 dpi, these signatures were more marked in the day 7 transcriptome. Flow cytometric analysis of microglia confirmed this at the cellular level; there was a greater proportion of cells expressing detectable MHC Class I on the surface of microglia from the brains of *PbA* infected mice, and increased expression levels on the cell surface at day 7. At this time, the transcriptome also showed upregulation of gene expression of proinflammatory cytokines/chemokines, and chemoattractant molecules. Together these data suggest a developmental change in microglia, of initial activation and proliferation, followed by functional maturation. Our study did not locate where in the brain this proliferation and maturation takes place, but Hoffmann *et al*.^32^ previously identified a restricted pattern of microglial activation in ECM in the olfactory bulb spreading along the rostral migratory stream, locations previously described for neuro-immunological cells^32^.

Upregulation of MHC class I, and production of chemokines would suggest a role in migration, antigen-presentation to, and activation of CD8 T cells, crucial players in the pathogenesis of ECM^33^. Indeed, CX3CR1+ cells (inflammatory monocytes, dendritic cells and microglia), have been observed close to the abluminal surface of blood vessels in mice infected with *PbA*, and associated with T cells^14^. It is unlikely, though, that this interaction is the priming event for CD8 T cells, as that is thought to take place already on endothelial cells^34^. However it is possible that a secondary activation and amplification of the CD8 T cell response following interaction with microglia contributes to additional brain injuries.

The transcriptome analysis strongly indicated of a role for Type I IFNs in the activation of microglia. Many of the genes involved in Type I IFN pathway were differentially expressed in the samples at both day 5 and day 7 of a PbA infection (Table S4). Although there was no evidence from the transcriptome of upregulation of Type I IFN gene expression in microglia themselves, it is possible that microglial cells are responding to external Type I IFN signal. In this regard, we confirmed that a microglial cell line responded to IFNβ *in vitro*, but not to PbA iRBC, by producing several of the chemokines detected by the microarray analysis.

The source of Type I IFN during ECM, which could be responsible for activation of microglia, is not clear. Many different cell types produce Type I IFNs. Transcription of Type I IFNs has been shown for pDCs and to a lesser extent cDCs^35^,^36^, and neutrophils have an Interferon transcriptional signature during *P. vivax* infection^37^. Most of these cells are not located within the brain; however with disruption of the BBB it is possible that cytokines such as IFNs access the brain parenchyma and thus microglia by this route.

In this study, examination of microglia from the *PbA* infected mice revealed Type I IFNs, as possible upstream regulators of MCH-I. This is compatible with previous reports showing upregulation of MHC-I molecules by Type I IFNs on thymocytes and lymphocytes^25^, and on cells of the central nervous system^38^. IFNβ has also been shown to stimulate microglia to produce of several chemokines, particularly CXCR3 ligands CXCL9, CXCL10 and CXCL11 and CCR2 ligands CCL2, CCL8 and CCL12^39^,^40^. In our study similarly, we found increased expression of genes encoding CXCR3 and CCR2 ligands (CXCL9, CXCL10, CCL8, CCL12) in the transcriptome of microglia from infected mice, especially at day 7, which was confirmed at the protein level in the supernatants of isolated microglia from infected mice after short-term *in vitro* culture. Cxcl10 is important for the pathogenesis of ECM^41^, and, upregulation of Cxcl10 during ECM has been recently demonstrated^42^.

Together, our transcriptomic analysis and the accompanying cellular and protein verification predicts a model in which microglia in the brains of mice undergoing ECM induced by *PbA*, are activated first into cell cycle and proliferation, and then to production of chemokines and upregulation of MHC-I via an external Type I IFN signal.

The consequences for ECM of activation of microglia are less clear. It is possible that their production of proinflammatory chemokines contributes to infiltration and activation of immune cells and thus amplification of the pathology. However, it has been shown that that global deletion of CX3CR1-expressing cells from day 3 of a PbA infection did not substantially affect the course of ECM^14^. The authors suggested that microglia and astrocytes might have an amplifying effect on cerebral inflammation. An alternative possible role for activated microglia could be an attempt to limit the damage to brain tissue by the inflammatory response taking place in ECM. Type I IFNs are known to have an effect on cell cycle and cell survival of immune cells, and to modulate some autoimmune diseases and cancers^43^,^44^,^45^, and relevant for ECM, is the observation that MHC class I upregulation by IFNβ in neurons and reactive glia may be beneficial to neuron recovery after brain lesions^38^. To determine unequivocally whether microglia have any causal effect in ECM, a cell-specific deletion of microglia would be the optimal approach, as well as a conditional knock out of the IFNRαβR in these cells.

In summary, our data set offers the possibility to mine the microglial transcriptome in the resting state and upon activation during ECM and to compare with other cerebral syndromes, or with other immune cell types in the brain, to understand better microglial biology and the role they might play in this severe malarial complication.

## Material and Methods

### Mice

Female C57BL/6 mice aged 6–9 weeks were obtained from the SPF unit of the Biological Research Facility at the Mill Hill Laboratory, Francis Crick Institute and were housed conventionally with sterile bedding, food and irradiated water. Room temperature was 22 °C with a 12 h light/dark cycle; food and water were provided *ad libitum*. The study was carried out in accordance with the UK Animals (Scientific Procedures) Act 1986 (Home Office licence 80/2538 and 70/8326), was approved by the MRC National Institute for Medical Research Ethical Committee and was approved by The Francis Crick Institute Ethical Committee. All surgery was performed under sodium pentobarbital anaesthesia, and all efforts were made to minimize suffering.

### Infection with *Plasmodium berghei* ANKA and Experimental Cerebral Malaria (ECM)

Mice were injected intraperitoneally (ip) with 10^5^ PbA infected red blood cells (iRBC) directly from an infected donor mouse. Parasitemia was monitored daily from day 3 post-infection by examination of Giemsa-stained thin blood films.

The appearance of neurological complications was score using the clinical signs typical of ECM, including ruffled fur, hunching, lost of equilibrium, partial paralysis, coma, lost of body weight and drop of temperature as described previously^46^. Body temperature of 31 °C or lower (measured by a rectal thermometer), indicates that severe symptoms was reached, and the mice were euthanized.

### Evans blue

To monitor the integrity of the blood brain barrier during experimental ECM, groups of control and PbA-infected C57BL/6 were injected intravenously with 0.1 ml of 2% Evan’s Blue dye in sterile phosphate-buffered saline (PBS) 6 days post infection^47^. The dye was allowed to circulate for 2 h, and then the mice were sacrificed, systematically perfused with 10 mL PBS and the brains were isolated and photographed. Brains were then kept in formamide at 37 °C and the absorbance (620 nm) was measured to detect the concentration of the blue dye.

### Isolation of cells from adult mouse brains

Mice were terminally anesthetized by intra peritoneal (ip) injection of 100 ul of Pentobarbital (10% W/V), perfused with 10 ml of cold PBS to remove circulating RBC and leucocytes. Brains were isolated in cold HBSS buffer (without Ca++ and Mg++), and enzymatically digested using a Neural Tissue Dissociation Kit (P) (Milteny Biotec) according to the manual. After tissue dissociation, myelin was removed by centrifugation over a Percoll Gradient. Briefly, the digested brain samples were resuspended in 4.5 ml of Percoll (GE Healthcare) 75% (in PBS1x) in a 15 ml tube; Percoll 37% (in red HBSS, company) was overlayed up to 14 ml. The tubes were centrifuged at 800 *g* for 20 minutes at 4 °C without brake. The surface ring on the top of the upper layer containing myelin and debris was aspirated by a vacuum pump and the ring between the phases 70% and 37% containing brain cells was collected, washed with PBS and labelled for subsequent cell sorting^48^.

### Flow cytometry and cell sorting

Isolated microglia were incubated for 15 minutes with Fc Receptor block (BD), followed by 1 h staining at 4 °C in the dark with Zombie UV™ Fixable Viability kit (Biolegend) and the appropriate combination of fluorophore conjugated antibodies: APC-conjugated anti-CD11b, PE-conjugated-CD45, APCCy7- conjugated-Ly6C, pacific blue-cojugated-H-2Kb (Biolegend). Cells were then washed and resuspended in PBS containing 2% FCS.

Cells sorting was performed on MoFlo XDP (Beckman coulter) and the target cell population was directly dispensed into TRIreagent (Ambion) and stored at −80 °C till RNA isolation, or in fresh Iscove’s Modified Dulbecco’s Media (IMDM) for the *ex vivo* culture. For the flow cytometric analysis, 50,000 to 100,000 events were recorded using BD LSR-Fortessa X-20 flow cytometer. Analysis was performed by FlowJo-X flowcytometric analysis software (Treestar).

### RNA isolation

Total RNA from approximately 10^5^ sorted microglia cells was isolated using the Ribopure kit (Ambion). Concentration of purified RNA was determined by Qubit fluorometric quantitation using the HS assay kit (ThermoFisher Scientific), and the quality analysed by Agilent 2100 Bioanalyzer. Samples with a RIN score above 8.50 were used for the next steps.

### Gene expression microarray

Total RNA (300 ng) of each sample was amplified using the Total prep RNA amplification kit (Illumina) following the manufacturer’s manual, and further quantified by Qubit fluorometric quantitation and analysed by Agilent 2100 Bioanalyzer.

Amplified cDNA (1500 ng) samples were then hybridized to microarray slides at 58 °C for 14–20 h with Illumina MOUSE WG-6 V2.0 Beadarrays according to the manufacturer’s protocols at the High Throughput Screening facility of the Francis Crick institute.

### Statistical analysis, PCA and hierarchical clustering

Illumina GenomeStudio software was used to subtract background and scale average samples’ signal intensity. Further normalization analyses were performed with GeneSpring 13.1.1 GX (Agilent Technologies). First, all signal intensity values less than 1 fluorescent unit were set to equal 1, log 2 transformed and normalised per chip using 75th percentile shift algorithm. Transcripts were further normalized to the median across all samples. Probes were first selected with if they were present (cut off 0.6).

PCA analysis was performed using probes without statistical filtering. Statistical testing was performed at d5 and d7 separately using independent unpaired t-tests followed by Benjamini-Hochberg multiple test correction. Probes with a *p* value of less than *0.005* and a fold change cut-off higher than 2 fold were considered statistically significant for further analysis. Hierarchical clustering was performed on the average of the normalized values with Pearson Uncentered (Cosine similarity) distance.

### Gene Ontology, Functional Enrichment analysis and Pathway mapping

Differentially expressed genes were classified into functional categories according to the Gene Ontology (GO) using DAVID (www.david.ncifcrf.gov). Enriched GO terms (p < 0.001) related to 3 main processes (biological function, cellular component and molecular function) were elaborated and organised in pie charts.

To investigate the pathways that contained the affected genes, a two-step approach was taken using ToppFun and GSEA for gene set enrichment analysis. The ToppFun analysis involved the mapping of the genes against the KEGG and Reactome databases to identify enriched gene sets, and the results were subsequently crosschecked with GSEA using the same databases.

Genes that were differentially expressed were mapped against the mouse pathways in KEGG using the *gage* package from Bioconductor with the mouse genome annotation.

### Quantitative real time PCR

cDNA was synthesized from 50 ng of total RNA isolated from sorted microglial cells using Omniscript RT kit (Qiagen). The resulting cDNA was diluted up to 2 ng of RNA per reaction, for quantitative PCR analysis. All reactions were setup in a total volume of 10 μl using 2x LightCycler 480 Universal Probe Master mix (Roche) and Universal ProbeLibrary probes (Roche) as specified by the manufacturer. Combinations of primers (Sigma) and probes are listed in Table S5. The PCR reactions were carried out using a LightCycler 480 (Roche); denaturation 95 °C for 5 minutes, 45 cycles of 95 °C for 10 seconds, 60 °C for 20 seconds, 72 °C for 1 second, followed by 40 °C for 10 seconds final cooling. The relative mRNA level was calculated using the comparative 2^−∆∆Ct^ method^49^ and normalized against HPRT level. Pearson’s correlation analysis was further used to correlate both RT-qPCR and microarray data using Prism6 (GraphPad), and the correlation coefficient and *p* value were calculated.

### Isolation of iRBC

Infected mice were terminally anesthetized by intra peritoneal (ip) injection of 100 ul of Pentobarbital (10% W/V). Blood was collected by cardiac puncture and added with 50 ul of heparin (300 U/ml) to avoid coagulation, diluted 1:10 with PBS and filtered with Plasmodipur filters to eliminated lymphocytes. After two washes with PBS, iRBC where separated from the rest of the blood by 60% Percoll gradient, counted and checked for their purity on a Giemsa-stained thin blood films.

### *In vitro* culture of microglia and measurement of chemokines

FACS-sorted microglia were seeded at a density of 10^5^ cells per well in 96-wells tissue culture plates in 100 ul IMDM culture media containing 10% FCS. Cells were cultured at 37 °C with 7% CO_2_ and saturated humidity for 6 hours.

BV2 cells were seeded at a density of 5 * 10^5^ cells per well in 6-wells culture plates in 1 ml IMDM culture media containing 10% FCS and left 1 hour to attach at the bottom of the wells. They then where treated with IFNβ (10^3^ U/ml) for 30 minutes and incubated with iRBC or RBC at a ratio 1/30 (cells/iRBC) for 24 h.

The supernatants were then collected and centrifuged at 400 *g* for 5 min at 4 °C to remove cells and debris, and stored at −20 °C. Supernatants were used for Cytometric bead array (CBA) measurement of chemokines with LEGENDplex (Biolegend) following manufacturer instructions. The statistical evaluation was performed on three independent experiments using Mann-Whitney analysis in Prism6 (GraphPad).

## Additional Information

**How to cite this article**: Capuccini, B. *et al*. Transcriptomic profiling of microglia reveals signatures of cell activation and immune response, during experimental cerebral malaria. *Sci. Rep.* **6**, 39258; doi: 10.1038/srep39258 (2016).

**Publisher's note:** Springer Nature remains neutral with regard to jurisdictional claims in published maps and institutional affiliations.

## Supplementary Material

Supplementary Information
