# Supplementary material for: Transcriptomic profiling of microglia reveals signatures of cell activation and immune response, during experimental cerebral malaria
Source: Sci Rep. 2016 Dec 19;6:39258. doi: 10.1038/srep39258 (PMC5171943; doi:10.1038/srep39258)
Supplement: Supplementary Information [file srep39258-s1.pdf]

Transcriptomic profiling of microglia reveals signatures of cell activation and immune response, during experimental cerebral malaria.

Barbara Capuccini[1,*], Jingwen Lin[1], Carlos Talavera-López[1], Shahid M. Khan[2], Jan Sodenkamp[1], Roberta Spaccapelo[3], and Jean Langhorne[1,*]

1) The Francis Crick Institute, London NW1 1AT, UK

2) Leiden University Medical Center, Albinusdreef 2, 2333 ZA, Leiden, Netherlands

3) Department of Experimental Medicine, University of Perugia, 06132 Perugia, Italy

*Corresponding authors: Barbara Capuccini and Jean Langhorne, The Francis Crick institute, Midland Road, London NW1 1AT, barbara.capuccini@crick.ac.uk and jean.langhorne@crick.ac.uk, Tel: +442037961498**Supplementary table S1: Differentially expressed genes at d5.**

Entrez Gene ID	ProbeID	Gene symbol	Fold change vs naïve
231655	4780253	Oasl1	562.69806
	2810612	Birc5	391.27356
71988	7050768	Esco2	382.88635
70218	2680010	3000004C01Rik	354.85516
16571	6660050	Kif4	287.88898
70218	1710368	3000004C01Rik	275.62695
	4730246	LOC226690	244.59027
	2190164	Ccnb1	236.2867
246728	3060450	Oas2	232.05135
	7100301	2810417H13Rik	219.29144
218977	4900215	Dlg7	198.97905
66442	2000270	Spc25	187.67982
269582	6980315	Clspn	181.98192
	2630066	Ckap2	175.79112
	7400215	Birc5	169.69556
70024	4220326	Mcm10	169.05167
16551	2360189	Kif11	165.70898
	5360129	Mki67	165.63124
	3140707	Tpx2	161.57355
18175	3170047	Nrap	160.49538
231655	240187	Oasl1	159.16177
237911	2070242	Brip1	151.91162
54141	2190064	Spag5	145.91838
14793	4920148	Cdca3	140.77196
71819	4850079	Kif23	140.17279
	380661	5730547N13Rik	139.85246
	1770349	Zfp276	131.78625
	4780360	Troap	127.040886
	7040475	2610019I03Rik	121.077415
	1580632	4921511I23Rik	118.213234
623474	610039	E130016E03Rik	116.6728
18973	4060441	Pole	116.4709
	1240446	Birc5	111.2811
217653	3850358	C79407	109.226364
23962	7050014	Oasl2	108.35412
20419	4280047	Shcbp1	104.15031
	1500446	Cdca8	102.97447
	6020722	Ccne2	102.247444
102920	6110646	Cenpi	101.91403
12449	4890292	Ccnf	101.12314
	4570458	Sn	99.43179
	3440725	Rrm2	93.56047
228421	3180164	Kif18a	92.33604
60411	4860600	Solt	92.18272

	3830017	5730547N13Rik	90.80971
67052	3390273	Ndc80	90.34923
17279	2650347	Melk	89.59565
	6130095	C030025P15Rik	82.70166
	3830328	Cdc25c	81.812965
19361	6480739	Rad51	79.30189
	4570440	2610019I03Rik	79.13615
27221	2030068	Chaf1a	78.98265
	1410070	Kntc1	78.83032
52033	2900300	Pbk	76.3822
	4780193	Calmbp1	76.21254
269582	2600019	Clspn	75.77537
	1050092	Serpina3g	75.30847
	5310209	2610021K21Rik	73.70003
67196	3190403	2700084L22Rik	73.50177
	1740554	scl0002624.1_576	70.703896
21973	4250403	Top2a	70.525604
	6590653	Irf7	69.78389
	4850324	1190002F15Rik	68.70011
74107	3710050	1200008O12Rik	67.92994
	60368	Slp	67.928276
	130348	Slp	66.872345
68713	4010019	Ifitm1	66.182945
	6420170	BC006779	64.16627
	430202	Top2a	63.499474
	4120452	2610019I03Rik	62.014538
	6370082	Rad51ap1	61.223118
17279	2140156	Melk	60.908543
	10161	Depdc1b	59.47352
	5360195	Sgol2	59.079838
70024	650121	Mcm10	58.730095
19361	110520	Rad51	57.845093
51944	5820193	D2Ertd750e	56.591263
269582	7570278	Clspn	55.945942
219114	4830687	F630043A04Rik	55.087727
	5290736	Kifc5a	54.96754
110033	4210246	Kif22	54.83493
231507	5860243	Plac8	53.18062
	6450259	Ifi205	52.384415
268930	7160689	Pkmyt1	52.240856
68743	1190201	Anln	51.89771
	1740491	2310007D09Rik	50.564243
	5420709	Rad51ap1	50.12598
233406	780475	Prc1	49.949154
	5490767	2500002A22Rik	49.44505
	6450634	1110017L21Rik	49.1867
229003	450706	BC006779	49.0742
11982	270180	Atp10a	48.936394

18824	1740379	Plp2	48.85782
	2190133	Ifi205	48.79241
	2650703	9830002L24Rik	48.718544
108912	1820274	Cdca2	48.689503
	2370168	Fanca	48.3662
66442	6620184	Spc25	48.29786
29870	1500148	Gtse1	48.097076
21877	7400142	Tk1	47.839474
	2350470	2610021K21Rik	47.53063
67629	4810047	Spc24	46.570045
	3840523	Kif23	46.217655
	5490181	4930547N16Rik	46.214584
	2760609	Bard1	46.09576
114564	6520392	Csprs	45.12898
	3840356	Rad51c	44.356777
231655	840259	Oasl1	44.3155
268930	3190343	Pkmyt1	44.282158
	4390170	Recql4	43.892036
14962	5260541	H2-Bf	43.567055
319148	4860209	Hist1h3c	43.183517
	540647	Cd69	42.887672
14793	1050170	Cdca3	42.579636
	380520	Stk6	41.99132
	4570689	Cit	41.43934
	580470	Tnfrsf5	40.83884
16571	1770168	Kif4	40.836857
12268	6510113	C4	40.38639
70385	2650521	2600001J17Rik	40.368267
	2630427	BC004701	40.12347
435565	6770259	LOC435565	38.63581
12268	7510243	C4	36.28671
12544	3400754	Cdc45l	35.295895
	2850717	H2-Q8	34.93239
20555	3520746	Slfn1	34.845673
66949	5220056	Trim59	34.064667
12531	2470360	Cdc25b	32.6228
17427	6280088	Mns1	32.589252
	540438	LOC328752	31.44799
	430333	5830426I05Rik	31.378687
18140	4560397	Uhrf1	31.308594
	6290390	Aurkb	31.226847
	130437	Mxd3	31.185476
21822	3890121	Tgtp	30.966557
21853	160703	Timeless	30.176195
	6980397	Kifc1	30.06568
68713	5960577	Ifitm1	30.048313
	3170487	F730004D16Rik	29.878084
23960	130598	Oas1g	29.21682

	2350095	Bub1b	29.011826
15951	7550093	Ifi204	28.723595
	3780474	9930016I07Rik	27.954042
	7400528	Ifi205	27.713778
15019	2750128	H2-Q8	26.31227
	1240538	P2ry14	26.307
217653	4640673	C79407	26.039589
100535	3120068	Oas1d	25.94827
23960	3890551	Oas1g	25.930876
106795	6330725	Tcf19	25.89013
72155	1690360	2610510J17Rik	25.737501
58185	5560474	2510004L01Rik	25.694077
70466	2450270	2610318C08Rik	25.592438
19366	7560373	Rad54l	25.512104
	4810184	Dlm1-pending	25.50052
107995	4610722	Cdc20	25.491926
24110	4610139	Usp18	25.02767
107995	4390228	Cdc20	24.927809
20684	1170110	Sp100	24.784704
	2320678	Cdc25c	24.63742
21877	3450010	Tk1	24.16144
	5820128	0610037M15Rik	24.160961
12534	6060379	Cdc2a	23.444088
15959	3460504	Ifit3	23.40348
	1990270	2610318C08Rik	22.509403
	520427	Plk1	22.199394
	2030026	Cdc6	21.908087
	3840575	Cd244	21.627584
	3890292	D630022O22Rik	21.210865
58203	1580634	Zbp1	20.905413
219131	1340193	Phf11	20.780283
23962	4830010	Oasl2	20.314072
15958	5270398	Ifit2	19.826252
	4830543	2310061N23Rik	19.707273
17279	4210671	Melk	19.63269
219131	6660327	Phf11	19.614595
23960	3890328	Oas1g	19.51171
	160041	Mmp25	19.162018
14469	6860600	Gbp2	18.953106
	1090382	Cdkn3	18.884884
	1410554	Tyki	18.880236
	3890682	2610036L11Rik	18.792707
109242	1340010	Kif24	18.72656
60530	670500	Fignl1	18.591211
	2940192	E130301L11Rik	18.377993
	2470730	2810417K24Rik	18.245657
	6580041	Ifi205	18.057266
	2900138	Mphosph1	17.879044

	3360253	Rad51c	17.66107
	4830386	Diap3	17.531418
102084	520040	AI451557	17.500185
16647	7210291	Kpna2	17.431828
110454	1690187	Ly6a	17.34583
66141	3840292	Ifitm3	17.21303
24110	2490452	Usp18	16.801826
12263	4210470	C2	16.723026
	2710703	E130306D19Rik	16.599558
	1190739	A630055A13Rik	16.511133
73804	1780543	Kif2c	16.318998
68298	4480070	Ncapd2	16.313654
53606	460221	G1p2	16.0889
72155	7650195	2610510J17Rik	15.747956
	2490653	LOC240327	15.672491
	6580379	P2ry14	15.054558
13555	60369	E2f1	14.726257
	10598	Cxcl9	14.709968
74747	130634	Ddit4	14.460221
68283	3360131	9530077C05Rik	14.176626
14190	2340242	Fgl2	14.135186
	5820709	LOC240921	14.129
	1090215	Sap18	14.08261
67775	2760577	Rtp4	14.017168
21939	4760241	Cd40	13.926463
	5960097	1700113I22Rik	13.857943
238673	2940632	Zfp367	13.7803
15945	3140209	Cxcl10	13.415448
15959	7510020	Ifit3	13.391039
	6020487	Cyp4f18	13.3548155
110749	7200270	Chaf1b	12.672548
432879	7330152	LOC432879	12.548698
24110	1580528	Usp18	12.35745
70218	6350367	3000004C01Rik	12.354938
	1090647	9830148G24Rik	12.350932
29856	620202	Smtn	12.306075
	6860196	1700021E15Rik	12.304326
243963	1440450	Zfp473	12.255787
208084	6940411	AI449441	11.957833
16881	4050711	Lig1	11.731945
	7200519	Cenpa	11.665831
240084	3180039	Cchcr1	11.664718
214901	1260022	Chtf18	11.655519
12236	5910528	Bub1b	11.510143
	1580356	Foxm1	11.308377
217946	4220139	BC006933	11.209746
	3890376	2610305J24Rik	11.047728
59027	2750189	Pbef1	11.006387

	6180504	C030025P15Rik	10.630138
211651	1940020	Fancd2	10.614785
15958	360041	Ifit2	10.603745
	1400220	LOC215405	10.409838
	5130059	Ifitm6	10.409609
	3940242	AA175286	10.3711815
14469	1400041	Gbp2	10.120155
67849	3310196	Cdca5	9.812839
329207	1500327	Gm817	9.693724
	380068	4732496O19Rik	9.680233
	2190215	Cxcl10	9.642974
	110259	Cdc7	9.626499
15270	1090327	H2afx	9.472496
	5900075	5730588I11Rik	9.457416
66336	20608	Cenpp	9.309379
	6450598	D11Lgp2e	9.280994
	3610528	LOC331239	9.274826
15953	1090139	Ifi47	9.20182
17216	1770612	Mcm2	9.0858
18610	3610332	Pdyn	9.01192
	4040095	H2-Q6	8.962184
	5720397	D11Lgp2e	8.9583645
	290575	Impa2	8.522373
17219	270379	Mcm6	8.392076
630499	4640348	EG630499	8.33847
	6350114	Ube2l6	8.326761
	3290292	Mx1	8.17586
	2650754	Ube2l6	8.080873
14056	1300035	Ezh2	8.024011
20847	6220594	Stat2	7.8547964
	6280221	Sp100	7.812571
16319	630634	Incenp	7.791611
625360	5670156	LOC625360	7.7538133
	4290739	Plekhk1	7.7275677
22137	5270544	Ttk	7.668121
80861	6330592	D11Lgp2e	7.667838
231832	3780113	BC019731	7.570831
80876	840349	Ifitm2	7.52459
	2480709	Foxm1	7.499896
	7150431	LOC237877	7.3081303
74016	4730131	Phf19	7.04683
19200	2360554	Pstpip1	6.9695625
77744	1340056	6720463M24Rik	6.692515
	4850689	A130019H11Rik	6.678477
	6280176	Mcm7	6.6618786
66140	990301	1110001A07Rik	6.6533284
	6220270	Mcm5	6.6288705
227659	7400286	Slc2a6	6.5806074

667977	270367	H2-gs17	6.543152
	1580309	Hap1	6.523125
	5560176	4930422G04Rik	6.476602
	5490386	D230036F23Rik	6.427278
	1110445	LOC380706	6.3832564
	7610129	8430438L13Rik	6.3544836
	7510408	Adora1	6.347123
12517	5700528	Cd72	6.3380733
	6290592	LOC226691	6.188622
19340	1470278	Rab3d	6.13619
56150	1410189	Mad2l1	6.081124
107995	1570754	Cdc20	6.0072384
	4290487	Mad2l1	6.0051785
	6760762	Sdc3	5.9295683
17219	3290437	Mcm6	5.9200473
	520278	Mx2	5.8848405
20556	4120307	Slfn2	5.795018
103737	2060730	Pex12	5.7301335
219132	2810040	D14Ertd668e	5.615501
16881	3060767	Lig1	5.5961924
16906	840673	Lmnb1	5.5866737
	4260528	Stat1	5.550217
56628	1710768	LOC56628	5.4894485
15024	870446	H2-T10	5.4878087
	510402	4833427B12Rik	5.4096622
	2230538	Cd44	5.3524323
12257	4670544	Tspo	5.3130355
97165	5290279	Hmgb2	5.297658
	7160133	Gas2l3	5.2026615
	830537	LOC269515	5.1953917
20133	6400348	Rrm1	5.139351
	3840279	4833427B12Rik	5.090257
	5960386	2810453L12Rik	5.0766726
	1780338	4833427B12Rik	4.985267
50878	160753	Stag3	4.9684772
22172	3830735	Tyms-ps	4.8521776
244183	4230500	A530023O14Rik	4.8214192
12257	2760544	Tspo	4.79256
	4010097	Map4k1	4.7830772
	6280521	A530060O05Rik	4.7688146
22171	2100026	Tyms	4.761206
	3940458	Rfc4	4.760301
	2320575	Mcm7	4.750655
12268	1010632	C4	4.703818
246256	830632	Fcrl3	4.702372
54563	1230703	Nup210	4.6882267
	6250600	Cdkn2d	4.655705
547343	1400132	LOC547343	4.654432

69550	2100139	Bst2	4.651943
12575	2070377	Cdkn1a	4.6513176
	4570669	2810475A17Rik	4.6471457
21354	5870093	Tap1	4.6280155
	1470605	D11Ertd759e	4.6154475
	70546	Hist1h2ag	4.582562
14972	6280026	H2-K1	4.570758
15944	5820608	Irgm	4.567409
13007	4780066	Csrp1	4.5516977
233876	5890600	C86302	4.533172
219132	520053	D14Ertd668e	4.492915
	7510768	Alms1	4.480006
	6100735	Lmnb2	4.439131
27280	1980021	Phlda3	4.345725
	160463	Lgals3bp	4.325593
22021	2120463	Tpst1	4.3107605
217430	5890523	Pqlc3	4.280392
	2510333	Ccl12	4.254782
12257	6270709	Tspo	4.228617
12575	7040491	Cdkn1a	4.1080008
19183	2900368	Psmc3ip	4.105546
27214	3930176	Dbf4	4.102268
	240725	Stat1	4.096473
	6590215	H2-T22	4.0960793
	2760368	4930524J08Rik	4.0581093
404710	5270717	Iqgap3	3.9945486
	3140731	Zranb3	3.9805925
19106	830762	Prkr	3.9803104
	1740427	Pml	3.967862
	5700019	Dscr1	3.964518
67037	3520162	Pmf1	3.9035442
	3460037	Fen1	3.8857737
15018	6940386	H2-Q7	3.8829565
66910	6550768	1110004B13Rik	3.8824103
	6220634	2810418N01Rik	3.8711207
22171	4810239	Tyms	3.8473623
	1710377	scl0002449.1_77	3.8451912
94094	610184	Trim34	3.8375728
	2320181	A330042I21Rik	3.8157623
	2650326	Pml	3.7510505
	6100286	scl0003903.1_2	3.7509995
12144	5960189	Blm	3.702935
	4040471	Skp2	3.7000594
242705	770162	E2f2	3.6865408
	7400612	Gcat	3.6840715
	430358	BC022145	3.6496854
	3990246	Ahcy	3.6341739
	2600671	BC066140	3.6304922

320148	2630433	B430306N03Rik	3.6288428
15013	5720048	H2-Q2	3.6151996
56417	3610730	Adar	3.6127155
21917	5390494	Tmpo	3.6026416
26362	7100577	Axl	3.6006958
78833	50446	2700085M18Rik	3.58313
94094	4290128	Trim34	3.5758214
242705	6550164	E2f2	3.5640287
	6100113	Hist1h2ab	3.5487769
	5390088	LOC327957	3.5443697
17219	3990243	Mcm6	3.5258489
	3140446	2310020F24Rik	3.5075839
110956	3170494	D17H6S56E-5	3.5060594
	50059	AI504432	3.5028028
12580	7150528	Cdkn2c	3.4952357
	5270475	Trex1	3.4327192
12419	3890519	Cbx5	3.4222398
	3440538	Comp	3.4183524
	4850669	Blm	3.4174294
	6100494	2610300B10Rik	3.3983343
547253	2970521	Parp14	3.3897967
327957	1190477	A430084P05Rik	3.3726218
12575	3130630	Cdkn1a	3.362913
	2230731	H2-T17	3.3515167
55932	1690475	Gbp4	3.3509989
	6350189	Trex1	3.349725
	780221	H2-T9	3.3308468
	3780736	H2-K1	3.3197384
	6450682	H2-L	3.292021
	4010288	4930599N23Rik	3.263419
	6940435	2310022K01Rik	3.2409334
	2600021	C330011F01Rik	3.2387397
319168	1470341	Hist1h2ah	3.2357116
18971	5220358	Pold1	3.2345538
319169	3130609	Hist1h2ak	3.2256048
101739	430204	Psip1	3.2193174
414801	2940612	BC063749	3.216898
	2490142	Ddx58	3.204791
	4730367	Ifi30	3.2008178
	990615	LOC239122	3.200462
67177	1050706	Ris2	3.1697319
102871	430079	D330045A20Rik	3.1551597
243771	1340050	Zc3hdc1	3.146364
	5340762	AW212394	3.1390858
	3420095	Whsc1	3.1265082
319165	3520717	Hist1h2ad	3.115046
	5900520	Gbp5	3.0711741
17217	2320368	Mcm4	3.0686355

	70348	Adar	3.0637124
	2360646	4930579G24Rik	3.0491064
14841	6420215	Gsg2	3.0468266
101565	3420521	6330503K22Rik	3.0295348
70454	2000139	Cenpl	3.019322
	610743	Nasp	2.9920003
	6250437	Ddx11	2.9897318
	1170402	B130065G19Rik	2.9879234
	1780452	Fbxw17	2.9794774
56452	6060376	Orc6l	2.9767556
	6760390	H2-L	2.959023
108670	2760274	2310046K10Rik	2.9193912
	620577	4930599N23Rik	2.9126694
56417	1780154	Adar	2.9054666
78658	2750114	B130055D15Rik	2.9048011
210106	7510743	Pols	2.901843
	3440615	Dscr1	2.892768
	3870706	AW540478	2.844576
	4290709	Ifi30	2.8440914
55932	60553	Gbp4	2.8426292
	3400491	Clic4	2.8323479
	3390735	Nasp	2.8069084
72140	3990255	2610507L03Rik	2.780156
	4120014	Ifi35	2.771492
74257	4880026	Tspan17	2.76344
380732	5960678	Gm885	2.7553382
	1990221	Ccl12	2.7304683
19650	4890291	Rbl1	2.7213686
72549	6250424	2700029E10Rik	2.7195938
	7650228	LOC380732	2.7056408
	1770754	5830411K18Rik	2.7024515
14760	3420326	Gpr19	2.6994576
	5290112	Rangap1	2.6608508
	1110543	Trim30	2.65025
	10167	LOC56628	2.6476412
74137	1770592	1200013B22Rik	2.6413262
15040	4010154	H2-T23	2.6281953
15040	7200100	H2-T23	2.597183
	1940338	H2-M3	2.5606103
17215	2360193	Mcm3	2.556915
101565	5080162	6330503K22Rik	2.5545757
16145	6290037	Igtp	2.552399
214444	110494	Cdk5rap2	2.5231538
17069	6550376	Ly6e	2.453925
319173	4250711	Hist1h2af	2.4521556
	4200202	6330500D04Rik	2.4188282
15896	7510452	Icam2	2.4179997
104806	1980341	Fancm	2.40774

66131	3990326	Tipin	2.3965726
	4810358	Dsip1	2.3932896
17215	7570014	Mcm3	2.3886893
67824	6450072	1110025F24Rik	2.3805692
	1230241	4631422C13Rik	2.3778486
	2970598	Map3k8	2.3416035
	1510121	BC003324	2.3383157
	6840021	Tuba6	2.3359573
329910	6250128	Acot11	2.314527
	4480102	H2afv	2.2890203
51788	3140500	H2afz	2.2864678
	5720626	Chc1	2.2620075
56045	3190577	Samhd1	2.2548714
	1710280	BC027246	2.2478027
	4480767	Dnmt1	2.2403398
320394	3170154	G630055P03Rik	2.2341466
	3840653	Sag	2.2322276
105837	1980079	Mtbp	2.220794
	1230681	LOC268569	2.215311
	5090181	Trrp2	2.2144773
69912	4730292	Nup43	2.2114968
103468	7040035	Nup107	2.2114663
	4490239	AI481105	2.2033606
	5870519	4631410M14Rik	2.1598518
76073	4150682	0610009F02Rik	2.158504
72075	5690717	Ogfr	2.1514513
16391	6520133	Isgf3g	2.150713
	2000541	2610207P08Rik	2.1228254
	4920598	Pik3ap1	2.1102242
227613	5890162	4930542G03Rik	2.1093855
14964	4060735	H2-D1	2.103427
103268	6220133	2410017P07Rik	2.1028826
212377	5290608	F730047E07Rik	2.1008997
	380215	Tor3a	2.081023
	6450253	5830484A20Rik	2.0580773
	2450064	Psmb9	2.0545812
16362	3360138	Irf1	2.0500505
52009	50609	Hn1l	2.0253005
	10402	Trim25	2.024748
12363	5290017	Casp4	2.0126648
60533	3870561	Pdcd1lg1	2.0046785
51788	6510575	H2afz	2.0031264
319845	160291	E130103I17Rik	-2.0002742
56504	20465	Stk23	-2.0004938
16985	380482	Lsp1	-2.0060742
	4920491	6720458D17Rik	-2.0211227
	2970273	1200014P03Rik	-2.0282977
	4290544	Nav1	-2.029888

235504	160553	Slc17a5	-2.032318
22268	4880433	Upk1b	-2.0381157
19400	3370482	Rapsn	-2.0416818
13537	1170521	Dusp2	-2.0420175
19400	4880056	Rapsn	-2.0431368
	4120615	A930024F17Rik	-2.0478303
	5890110	Cbfa2t3h	-2.0522144
545007	1990021	LOC545007	-2.0700693
	1050300	Rgs2	-2.0700793
66569	4280274	2610020H15Rik	-2.080473
	4260564	Ppgb	-2.0897908
215690	4850731	Nav1	-2.0990152
243813	4210300	Leng9	-2.1017935
109245	2690524	9430028I06Rik	-2.1122112
	4070025	Slc12a2	-2.119844
14697	830427	Gnb5	-2.1302993
	540543	Tm7sf3	-2.1366732
	7400348	Arid3a	-2.1454322
	4560070	Myo18b	-2.1507146
13641	1400152	Efnb1	-2.15123
	4860747	B230363H02Rik	-2.1644971
19376	1780025	Rab34	-2.171903
	70386	Satb1	-2.1719966
	4900372	4921531D08Rik	-2.1847236
	1710333	Zdhhc14	-2.1948388
	1850437	Armc3	-2.1948707
23945	5310328	Mgll	-2.1989791
	6450520	7530408C15Rik	-2.1999533
	6940196	Aatk	-2.2241676
237052	4570468	Tceal1	-2.224956
545013	6180379	LOC545013	-2.2506144
	840477	scl0015365.1_6	-2.25334
12345	2030215	Capzb	-2.254229
	4830220	Epn2	-2.259134
	2630364	1110021L09Rik	-2.260399
	1430368	St6gal1	-2.2709906
	1240465	A130020K16Rik	-2.2821946
83679	6100612	Pde4dip	-2.2866604
52668	160437	D12Ertd647e	-2.2922044
	6380201	Nav1	-2.296743
	6420373	2810474C18Rik	-2.3001792
18670	7210136	Abcb4	-2.3056488
52668	1110280	D12Ertd647e	-2.309649
17826	5720538	Mtvr2	-2.317834
12479	2490577	Cd1d1	-2.3184435
	6660181	Peg13	-2.3247225
	5870044	1110012N22Rik	-2.3322668
	4780040	Abcc3	-2.339223

69032	2340255	Lyzl4	-2.3454106
13650	3840598	Rhbdf1	-2.3529408
	3460110	5031436O03Rik	-2.3533027
74202	4260195	Fblim1	-2.3574107
	7150356	Hist2h2aa1	-2.3662019
	670440	Rgs2	-2.3817487
	3120129	Gprasp1	-2.3840296
109711	4590136	Actn1	-2.3856559
407831	5360750	BC054438	-2.390279
	5820196	2310040B03Rik	-2.3965101
	5900575	Il6st	-2.3983967
	2480041	2010320M18Rik	-2.406179
17179	1940646	Matk	-2.408773
	2630086	9630015D15Rik	-2.4223473
52666	290348	D10Ertd610e	-2.4268925
	1340224	BC044804	-2.432248
109652	2370148	Acy1	-2.4812126
109652	1510669	Acy1	-2.4880714
229499	6980440	A230020G22Rik	-2.490682
	6980292	scl00001.1_0_REVCOMP	-2.516428
	5700681	6330400D04	-2.519962
	270601	1700110I01Rik	-2.5328107
	5290441	A730063M14Rik	-2.5495243
65112	3420544	Tmepai	-2.5505898
	6980630	Fhit	-2.5622401
70882	1440706	Armc3	-2.56744
	20255	2900026A02Rik	-2.5767481
	4120286	F630025I20Rik	-2.596889
229499	2490066	A230020G22Rik	-2.6237798
	1940215	2700046G09Rik	-2.6783268
	520692	E130115E03Rik	-2.6837754
	5810598	Col6a3	-2.7084057
17179	3440397	Matk	-2.7228594
	1030730	scl0002786.1_3	-2.7271452
	3290156	2310047K21Rik	-2.7570727
	2630605	F730031O20Rik	-2.7588706
	5050066	Ppm1l	-2.770062
	5890097	Sema4b	-2.7841656
	6420750	Sall2	-2.8113825
11732	3800670	Ank	-2.8429902
	3060600	Rab6b	-2.8640473
	5720091	C330013E15Rik	-2.8717666
12458	1010132	Ccr6	-2.8861675
	3440224	E130115E03Rik	-2.8952782
105193	1710736	Nhlrc1	-2.9281452
17965	3850255	Nbl1	-2.9304714
102657	5860327	6030411F23Rik	-2.9486904

16643	4890279	Klrd1	-2.951237
21928	1070630	Tnfaip2	-2.9553328
320333	1580386	D830030K20Rik	-3.0024807
	20615	Ppm1l	-3.0160806
	1850315	Sox4	-3.1069684
21935	2940482	Tnfrsf17	-3.1834717
	5570132	Ppm1l	-3.2246766
	6650477	Ian6	-3.2702284
	940025	LOC384472	-3.2945483
	6980609	6430590I03Rik	-3.3038375
14369	6020424	Fzd7	-3.3285582
654812	6520451	Angptl7	-3.3493447
	1940040	4931406O17Rik	-3.4370441
16644	4290521	Kng1	-3.478689
	2100468	Tlr5	-3.4942641
54368	4280414	Gp9	-3.531131
	580259	Catnd2	-3.5795825
	3140397	Gprasp2	-3.7651544
117590	6400360	Asb10	-3.971728
544988	610091	LOC544988	-4.0657215
	4780201	A830087P12Rik	-4.3517666
	610685	A130039H17Rik	-6.361968
	4200546	Adcy8	-6.4349313
	3170692	Slc5a10	-6.48146
	2650010	Cadps	-6.834843
70925	4290154	4921511I16Rik	-10.063032
	3290725	Rgs11	-11.553921
	150209	A430006M23Rik	-11.555361
	290193	LOC239618	-12.428441
545015	3310195	2610042L04Rik	-34.846573

Supplementary table S2: Differentially expressed genes d7.

Entrez Gene ID	ProbeID	Gene symbol	Fold change vs naïve
	3420139	Il1r2	1738.9353
	1050092	Serpina3g	1178.2552
	3170487	F730004D16Rik	624.3466
	540647	Cd69	414.19824
18175	3170047	Nrap	394.08554
	4730246	LOC226690	378.60544
231655	4780253	Oasl1	376.06052
13034	2140639	Ctse	363.12418
229595	130192	Tsrc1	349.7525
53608	7200025	Map3k6	289.86234
110168	2750543	Gpr18	289.33148
246728	3060450	Oas2	246.03413
	6400719	Saa3	245.93185
	7400528	Ifi205	243.71439
72054	2120392	Cyp4f18	243.066
	6450259	Ifi205	234.6777
	7560307	Irg1	205.81625
231655	240187	Oasl1	199.63985
	5340577	Xdh	191.93933
	60368	Slp	189.24611
14469	6860600	Gbp2	185.7916
68713	4010019	Ifitm1	181.21935
	130348	Slp	175.14546
	4540082	BC049975	162.00958
	7650048	Fpr-rs2	160.59178
	5820709	LOC240921	157.56786
	7550156	Ccnb1	157.49164
	1710619	Ifi205	149.37787
58217	4280504	Trem1	141.02757
	10598	Cxcl9	137.17833
100034251	5360370	LOC100034251	124.585785
	2190133	Ifi205	123.875114
70218	2680010	3000004C01Rik	123.54543
	5360082	scavenger receptor	123.53146
	4810184	Dlm1-pending	122.84565
	580470	Tnfrsf5	122.406746
20555	3520746	Slfn1	121.54738
58185	5560474	2510004L01Rik	120.90678
433470	7160047	AA467197	117.978874
	4570458	Sn	116.352745
231655	840259	Oasl1	113.63243
	3840575	Cd244	113.0421
18106	4540064	Cd244	112.13112
12268	2350291	C4	108.33983
	5490026	Emilin2	107.48486
545384	1690292	BC094916	106.09906

	5290546	Ctse	105.12385
	1230639	F10	104.386406
246707	5910164	Emilin2	103.78914
	6590653	Irf7	101.734886
16790	4150193	Anpep	97.24225
12268	6510113	C4	93.57881
14469	2100068	Gbp2	91.271385
	2810612	Birc5	90.84561
75750	4060100	8430417G17Rik	90.55123
	130288	A630085K21	84.78844
18712	5860039	Pim1	82.135574
74568	630025	Mlkl	82.00036
	3990711	9930022F21Rik	80.215256
20716	2450347	Serpina3n	80.012634
15951	3060554	Ifi204	79.68101
15958	5270398	Ifit2	78.73005
	2190164	Ccnb1	77.84311
12268	7510243	C4	77.391754
	4210239	Slc4a8	75.782936
15953	1090139	Ifi47	73.65926
	4920019	scl0003154.1_1	73.164566
15958	360041	Ifit2	72.548454
226421	2940176	5430435G22Rik	71.47891
80885	5700161	Gpr109a	70.25981
15951	7550093	Ifi204	68.763565
	7210184	scl000868.1_2	68.13868
	6020487	Cyp4f18	68.06434
	1580008	Aim1	67.17701
	3780474	9930016I07Rik	66.87922
16421	830543	Itgb7	66.24978
231507	5860243	Plac8	63.796135
	5670240	Map3k6	61.7045
	1500446	Cdca8	58.5363
	1660184	Ifi205	57.770176
70218	1710368	3000004C01Rik	57.555767
16169	6400270	Il15ra	57.363308
382045	4890092	Gpr114	56.143127
20343	5360707	Sell	55.43505
	6420170	BC006779	54.923664
16571	6660050	Kif4	53.191395
229003	450706	BC006779	52.501328
68713	5960577	Ifitm1	51.50538
	6940398	LOC270152	50.494034
	160041	Mmp25	50.069683
	430202	Top2a	49.045677
15959	670670	Ifit3	48.86549
	4890689	Oas3	48.21454
77037	5870487	1110025G12Rik	47.907784
	2190215	Cxcl10	47.90023
	1240538	P2ry14	46.20078
	7400215	Birc5	44.007782

	2630066	Ckap2	43.676556
219131	6660327	Phf11	43.559006
	3290292	Mx1	42.183247
102084	520040	AI451557	42.0044
14262	6020441	Fmo3	41.94585
	830072	Lilrb4	41.883453
219131	1340193	Phf11	40.53121
15959	3460504	Ifit3	40.514084
	2370630	2610204M08Rik	40.457348
	940180	1500004A08Rik	40.194153
14967	5050451	H2-D4	39.440598
16541	5690500	Napsa	39.22705
15945	3140209	Cxcl10	39.11977
16174	430446	Il18rap	38.17255
14469	1400041	Gbp2	37.915585
16012	7380603	Igfbp6	37.524082
	610324	Cd3d	37.4796
19340	1470278	Rab3d	37.32802
18610	3610332	Pdyn	37.00595
16169	5360678	Il15ra	36.09149
57444	540411	Isg20	35.519783
320832	5090491	Sirpb1	35.38076
66141	3840292	Ifitm3	34.70865
	3830328	Cdc25c	34.69117
12263	4210470	C2	34.474133
23962	7050014	Oasl2	34.468334
	3840523	Kif23	33.86732
	1410554	Tyki	33.596855
	6580379	P2ry14	33.438328
71819	4850079	Kif23	33.41603
23962	4830010	Oasl2	32.598
57746	6020187	Piwil2	32.320778
	1240446	Birc5	31.8259
	3180438	2010317E24Rik	31.57636
58203	1580634	Zbp1	31.169638
	160392	Cxcr4	31.085121
217430	5890523	Pqlc3	30.989872
	4050037	2310051E17Rik	30.516329
	4830273	Slc4a8	30.374073
20304	1690768	Ccl5	30.106688
16169	4540403	Il15ra	30.008772
	7040195	Gpr83	29.785194
	540438	LOC328752	29.579683
	5820128	0610037M15Rik	28.806526
15959	7510020	Ifit3	28.26784
	270010	Etv4	27.975542
13601	1710242	Ecm1	27.507017
20684	1170110	Sp100	27.380056
	2850717	H2-Q8	27.256311
	6250148	Dpep2	26.999645
	7200037	Adam8	26.899242

	4830543	2310061N23Rik	26.670538
13587	5870605	Ear2	26.616695
21822	3890121	Tgtp	26.397688
	3940242	AA175286	26.321419
	4810324	Serping1	26.261782
14190	2340242	Fgl2	26.0683
16497	6130471	Kcnab1	25.786854
29870	1780719	Gtse1	25.688337
217430	7650767	Pqlc3	25.38263
109979	5890338	Art3	25.015984
	650717	Lilrb4	24.588425
53606	460221	G1p2	24.55848
14744	5670687	Gpr65	24.491701
244183	4390594	A530023O14Rik	24.270988
	4730524	5430435G22Rik	24.24874
227659	7400286	Slc2a6	24.00264
228421	3180164	Kif18a	23.589624
	6280521	A530060O05Rik	23.228249
18726	5870113	Pira3	23.194569
327954	2900747	Dnahc2	23.014349
384009	3940639	Glipr2	22.771723
233406	780475	Prc1	22.764912
73804	1780543	Kif2c	22.484962
	3420433	Il12rb1	22.17348
80876	840349	Ifitm2	22.117163
20359	1470431	Sema6b	21.456257
	5080326	S100a6	21.447649
	4880020	2410187C16Rik	21.302517
20307	2480296	Ccl8	21.234295
16541	4830551	Napsa	21.201355
	1090647	9830148G24Rik	21.089972
	4070148	Ear1	21.07106
24110	4610139	Usp18	20.82286
	2490653	LOC240327	20.378166
	7400392	Sepw1	20.134321
	7040475	2610019I03Rik	20.124168
	3800136	Sepx1	20.08394
67775	2760577	Rtp4	20.039913
59027	2750189	Pbef1	19.794659
	6510241	LOC381329	19.725481
16601	4210327	Bteb1	19.36106
170741	4570204	Pilrb	19.349173
21853	160703	Timeless	19.271843
	6590064	Mrpl15	18.818035
	2030382	A930023F12Rik	18.803095
16169	6650142	Il15ra	18.628473
18793	5720497	Plaur	18.598827
	2230538	Cd44	18.14901
	4260220	LOC269531	18.10373
	1990397	A630047J05Rik	18.053263
	3890682	2610036L11Rik	18.032915

	2650703	9830002L24Rik	17.911345
	6590239	Cd3e	17.803812
74107	3710050	1200008O12Rik	17.48792
12268	1010632	C4	17.380741
	4570722	Arhgap15	17.371712
	7160241	Cybb	17.363625
	2350095	Bub1b	17.307188
	6580139	Itgp	17.305918
74481	7160246	4933430F08Rik	17.218475
15019	2750128	H2-Q8	17.208536
16859	2350537	Lgals9	16.909136
24110	2490452	Usp18	16.886606
	1440139	D11Lgp2e	16.613499
217430	3180152	Pqlc3	16.34599
327957	1190477	A430084P05Rik	16.291286
107995	4570088	Cdc20	16.014475
	7380324	scl000868.1_2	15.95385
16497	2570608	Kcnab1	15.946501
14793	1050170	Cdca3	15.603108
	4040095	H2-Q6	15.550358
15944	7380221	Irgm	15.520722
20847	6220594	Stat2	15.200038
24110	1580528	Usp18	15.180186
237256	6510142	D730019B10Rik	14.946174
16854	510474	Lgals3	14.723739
	1400220	LOC215405	14.715667
23960	3890328	Oas1g	14.694639
56620	5360056	Clec4n	14.434021
22352	1690091	Vim	14.369492
23961	5550010	Oas1b	14.200897
23960	130598	Oas1g	14.182916
	4850689	A130019H11Rik	14.169334
19200	2360554	Pstpip1	13.971952
	870221	Oas1a	13.947911
71712	5270100	1200002N14Rik	13.89088
	4220162	Stx11	13.698195
60533	3870561	Pdcd1lg1	13.669132
	1820164	Zbtb16	13.595215
107995	4610722	Cdc20	13.5548115
20359	2600561	Sema6b	13.525599
16574	3190639	Kif5c	13.440753
	1090382	Cdkn3	13.358812
110454	1690187	Ly6a	13.109668
74747	130634	Ddit4	13.0422535
68278	2940685	Ddx39	12.985363
	6450634	1110017L21Rik	12.865931
	4640754	F730045P10Rik	12.845463
	240491	Osm	12.822292
70466	2450270	2610318C08Rik	12.7789135
	3360273	A630059M09Rik	12.772151
217946	4220139	BC006933	12.735246

246256	830632	Fcrl3	12.401696
	3520142	Rog	12.352537
	270193	Tnfsf13b	12.3504
	5390088	LOC327957	12.330446
	6520193	Dnase1l3	12.29059
	6760669	Mafk	12.191158
80861	6330592	D11Lgp2e	11.935271
	520278	Mx2	11.761493
	6130682	BC003281	11.732226
	5720397	D11Lgp2e	11.684268
18301	2630008	Fxyd5	11.543703
	5820767	4930477M19	11.300333
219132	2810040	D14Ertd668e	11.199702
208104	2030520	B930074I24	11.182083
	6770195	Ii	11.144681
	6450598	D11Lgp2e	11.105779
547343	1400132	LOC547343	10.923836
	5860053	LOC381287	10.91014
	3780102	1810049K24Rik	10.704086
	1340500	AW125391	10.67308
15944	5820608	Irgm	10.640523
12575	2070377	Cdkn1a	10.639942
21354	5870093	Tap1	10.6270895
	6290592	LOC226691	10.529066
	1710040	LOC328833	10.462428
	3360338	Ii	10.235244
93695	4670228	Gpnmb	10.058177
99526	510400	Usp53	10.054506
	4900167	1810054D07Rik	9.985916
414801	2940612	BC063749	9.948042
	4610433	Prg	9.912737
	1110445	LOC380706	9.774183
15016	6980075	H2-Q5	9.767257
	6370333	4933424C13Rik	9.762396
	2680066	LOC245884	9.753611
18301	4780196	Fxyd5	9.752572
81703	5860717	Jundm2	9.740368
	2490328	Pml	9.696
20556	4120307	Slfn2	9.694468
	6650070	Pscdbp	9.66911
23961	3180445	Oas1b	9.617245
12575	7040491	Cdkn1a	9.581253
	7200093	4930412M03Rik	9.531002
	1660056	Ipas	9.453965
	6760762	Sdc3	9.253004
11837	5570093	Arbp	9.218644
	4010343	Sepx1	9.167709
12575	3130630	Cdkn1a	9.072537
667977	270367	H2-gs17	8.926894
	4880639	2810021O14Rik	8.896405
219132	520053	D14Ertd668e	8.892437

	3840521	Nfil3	8.86413
	7000059	1300013D05Rik	8.84362
22021	2120463	Tpst1	8.743125
	4830538	Iqgap1	8.7241125
232925	380148	Igfl3	8.671658
	3140446	2310020F24Rik	8.646372
	3290707	Tnfsf10	8.595983
	4120687	Glipr2	8.571342
21816	6270040	Tgm1	8.562749
27052	5700538	Aoah	8.553813
	3710161	Crk	8.425027
	4730440	D4Ertd432e	8.296131
56045	3190577	Samhd1	8.289592
26362	7100577	Axl	8.283421
14229	4850176	Fkbp5	7.887502
18413	6270600	Osm	7.8225384
12257	6270709	Tspo	7.808455
	5560437	6530401N04Rik	7.735415
	160463	Lgals3bp	7.654424
12257	4670544	Tspo	7.6079307
12257	2760544	Tspo	7.592547
	7200519	Cenpa	7.571469
	2510333	Ccl12	7.570478
	50059	AI504432	7.5653105
14538	1070242	Gcnt2	7.558873
	3830377	Iqgap1	7.5233083
	3460296	Anxa2	7.484685
	4250059	Notch4	7.4629498
	4540477	C920004C08Rik	7.3072567
	2850600	Alkbh	7.294417
18724	4280187	Pira11	7.216983
	6380669	Notch4	7.143372
15024	870446	H2-T10	7.133779
	7100592	Crk	7.1114135
22321	6840408	Vars	6.9417734
110749	7200270	Chaf1b	6.9409776
59027	1780600	Pbef1	6.9394655
	2370707	Cpeb2	6.89005
	2320403	Gch1	6.8550844
14538	6270739	Gcnt2	6.8463273
	5490187	Prg	6.8226147
	5910070	1700029M20Rik	6.6616583
19652	460100	Rbm3	6.6158233
56628	1710768	LOC56628	6.61271
	3400491	Clic4	6.604865
	6980685	Zfp26	6.595139
18787	4850563	Serpine1	6.589715
	990615	LOC239122	6.5531454
	6650278	2610507B11Rik	6.503366
380753	1660615	Atxn7l1	6.502496
16362	3360138	Irf1	6.442806

14972	6280026	H2-K1	6.434294
	7210608	Rnf149	6.3332925
	6250674	LOC211193	6.326254
	6350114	Ube2l6	6.2942476
	6280221	Sp100	6.2532887
	2260431	BC003314	6.239597
	4480685	H2-T22	6.1884785
	4540291	Klf13	6.0881124
	5270475	Trex1	6.081574
	6590215	H2-T22	6.0551276
	1990221	Ccl12	6.0475936
	2970274	2610029J22Rik	6.021704
	4220273	5830458K16Rik	5.912246
18768	110593	Pkib	5.906133
16188	2600240	Il3ra	5.8800983
	2030056	C130032L16Rik	5.872933
630499	4640348	EG630499	5.862693
236904	1780356	Klhl15	5.8315563
	460072	Csf2rb1	5.8219905
208727	1240433	4932408F19Rik	5.769556
	7330424	D19Ertd678e	5.746041
	780274	Auh	5.6847887
	1470605	D11Ertd759e	5.6647997
	6350189	Trex1	5.6476965
55932	60553	Gbp4	5.602284
53313	60288	Atp2a3	5.5642796
13367	7000390	Diap1	5.56363
20527	460327	Slc2a3	5.535356
16319	630634	Incenp	5.524906
	1050672	Dab2	5.510707
231532	3800528	Arhgap24	5.486095
	6270292	4921524J06Rik	5.4849505
22688	4490333	Zfp26	5.457933
	10228	LOC384525	5.452973
	6770674	LOC381579	5.428852
	2650326	Pml	5.3893933
	4150369	Daxx	5.3743515
69710	770538	Centd2	5.3463616
	2970598	Map3k8	5.332027
	6450682	H2-L	5.321051
56738	60600	Mocs1	5.3052626
16362	3400288	Irf1	5.3000603
	6250600	Cdkn2d	5.2991123
	7040619	Clic4	5.292379
320024	6960369	Aadacl1	5.277836
55932	1690475	Gbp4	5.2117867
74137	1770592	1200013B22Rik	5.1964893
71778	6660707	Klhl5	5.190814
19386	2630750	Ranbp2	5.141782
	830537	LOC269515	5.1135335
	5560349	2310015N21Rik	5.0919943

	5570020	1700027M01Rik	5.066368
	5670164	Adfp	5.0219064
231713	6960170	C330023M02Rik	5.020838
22321	1580044	Vars	5.0135007
231713	2360709	C330023M02Rik	5.00915
	7210349	Apobec3	4.97707
493809	1260020	Taar3	4.9595256
102595	7330392	AI840980	4.956658
	6370204	Tagln2	4.940986
12046	3520735	Bcl2a1c	4.9259906
	2630379	BC048355	4.883624
13132	1050609	Dab2	4.8824854
229900	6330692	Gbp6	4.8516703
15018	6940386	H2-Q7	4.8510656
	3130750	Ung	4.7990093
	580438	2810431I02Rik	4.7805233
69710	5720594	Centd2	4.754466
11898	7560338	Ass1	4.735025
	1170181	Cd52	4.7019424
78781	520600	Zc3hav1	4.673763
	6760390	H2-L	4.614608
	1850068	Pk3	4.6047325
	6480653	AI481105	4.596737
15013	5720048	H2-Q2	4.595039
	580142	Zfp106	4.5948944
	580746	Rasgrf1	4.5899887
	460184	9430029L20Rik	4.543333
15902	4490500	Id2	4.5132313
	2470356	Cox6a2	4.510886
71839	430037	1700012B18Rik	4.5064573
	6560431	B630009B09Rik	4.481977
	380215	Tor3a	4.4670405
214639	940129	4930486L24Rik	4.463845
56417	1780154	Adar	4.4280953
	1580561	C230091E20Rik	4.4277625
56075	5220110	Pdss1	4.4270973
	2650754	Ube2l6	4.388617
	5870563	AW540478	4.3441715
15040	2350181	H2-T23	4.328157
15902	1030519	Id2	4.317474
	780221	H2-T9	4.3108706
17158	3400050	Man2a1	4.3039985
243771	1340050	Zc3hdc1	4.2973847
	6270465	Rasgrf1	4.246866
14964	4060735	H2-D1	4.2292175
18626	20475	Per1	4.2241583
	2680687	Zfp106	4.2235045
	520025	2010002N04Rik	4.2208266
	3140377	scl0002116.1_6	4.216004
	270711	Galgt1	4.200215
	1780452	Fbxw17	4.195366

	4490239	AI481105	4.1942677
	2230731	H2-T17	4.186023
12696	6980576	Cirbp	4.179338
	2940138	Ctla2b	4.176888
	1010327	Tor3a	4.164545
15040	4010154	H2-T23	4.1311593
13367	3940021	Diap1	4.117726
17913	7570494	Myo1c	4.107861
68949	540138	1500012F01Rik	4.103646
108670	4880747	2310046K10Rik	4.0958786
15016	3130240	H2-Q5	4.091906
68591	6480201	Mocos	4.0858827
	870070	scl0003020.1_1	4.064895
	2450577	1810054D07Rik	4.0627494
73094	3310474	Sgip1	4.048459
	3850324	LOC381010	4.047519
12608	4230348	Cebpb	4.0453777
	3780736	H2-K1	4.0449452
	1710136	9626100_224	4.0164986
16362	6660634	Irf1	3.9968076
75316	5220687	Josd3	3.99293
	4480543	Mtf1	3.979722
	10167	LOC56628	3.9793358
380732	5960678	Gm885	3.971681
71361	4490452	Amid	3.9578784
	240468	Spint1	3.929983
15040	7200100	H2-T23	3.8949783
	6520431	Adar	3.8893309
	6510382	1810032O08Rik	3.8842642
231532	6550470	Arhgap24	3.858585
114644	1850674	Slc13a3	3.815194
	7210711	BC008150	3.8142629
	4120014	Ifi35	3.7912788
18626	2570037	Per1	3.7906425
13664	2850487	Eif1a	3.7673638
547253	2970521	Parp14	3.7473128
216161	6220037	BC019206	3.699811
	6270296	Slc9a3r1	3.690953
	2490142	Ddx58	3.67007
22321	4200543	Vars	3.6588008
28088	5570296	D10Wsu52e	3.6514256
	4490475	Flna	3.6510925
230279	2370669	6330416G13Rik	3.6317244
215474	110433	Sec22c	3.6037812
17748	5220279	Mt1	3.593315
	2810735	Cacna1s	3.581669
239647	2120187	BC038822	3.496812
	870593	9626100_15	3.4861646
	2340543	Tnfsf13	3.4842424
	5220220	9626958_317	3.4706616
	2710092	9030624G23Rik	3.4673681

231712	3440037	Trafd1	3.4356985
231712	3800068	Trafd1	3.4210925
	7330719	2010012F05Rik	3.4162972
114644	6980097	Slc13a3	3.4146807
	4730367	Ifi30	3.4139001
213233	6480131	Tapbpl	3.4079473
72056	150066	1810055G02Rik	3.3965676
	7610402	LOC234582	3.3958387
	3180754	Acbd4	3.3927968
	540603	Rbms1	3.3911402
100608	3180403	AI326906	3.357085
	5890553	Lss	3.347595
	2760468	BC062185	3.3268628
	1110131	LOC242051	3.3249
12363	5290017	Casp4	3.2787597
	3440021	Csf1	3.2767231
	4290709	Ifi30	3.2754958
77056	2490465	Tmco4	3.2589746
	450368	Rgs1	3.257137
12363	4290180	Casp4	3.246286
	1940338	H2-M3	3.2418087
	20692	2010107H07Rik	3.2273057
	7610743	Cnr2	3.2212815
12517	5700528	Cd72	3.2154794
16402	2070474	Itga5	3.2139559
	870243	Ldlr	3.2067897
	1440594	Fnbp4	3.1955419
67171	1440639	Tmem77	3.1805558
	10402	Trim25	3.1765685
	7150367	St14	3.162389
56878	10500	Rbms1	3.1555548
93692	4050112	Glrx	3.1493409
	5360575	LOC207695	3.1329749
170942	4880187	Erdr1	3.1159425
	1580754	2610014I16Rik	3.0976145
	5130037	C130074J06Rik	3.0933602
23833	6290768	Cd52	3.0908515
16859	430132	Lgals9	3.0874145
	4830072	Rgs1	3.0789595
208263	1010386	MGC6357	3.078458
	2760615	1500012F01Rik	3.076689
	3440685	Spata6	3.0759506
212547	6520154	BC027231	3.0737963
23790	3370195	Coro1c	3.0562103
	1240471	Hmgn2	3.0396543
19165	990682	Psen2	3.0393918
	4830735	E430031D18Rik	3.0374937
	3400452	LOC383706	3.0356023
	270717	BC023741	3.0338452
	3180025	Ms4a6d	3.0262704
	60152	LOC328625	3.0164156

	5960048	scl0002018.1_19	3.0050728
	1170195	BC021438	2.997795
	4040037	Tpm4	2.9914114
	3460037	Fen1	2.98707
	4290215	Bmf	2.9859498
19186	940674	Psme1	2.9852178
	6290193	2510010K19Rik	2.978314
	6420554	1810032O08Rik	2.954493
107734	580161	Mrpl30	2.9465709
	2100730	F630107D10Rik	2.9456155
	7100309	1700010A01Rik	2.945505
52502	7380364	Carhsp1	2.9209874
	4050609	Vti1a	2.9092226
70155	5720440	Ogfrl1	2.896157
77031	2340707	Slc9a8	2.8901064
	6960497	Chka	2.8888726
	770273	BC013712	2.8837738
	6900598	2310007G05Rik	2.8704562
74244	5310746	Apg7l	2.863637
	5670398	Trim21	2.8592765
	1110088	Apobec1	2.8509338
	3850041	Ddx46	2.8455956
	7550445	Acbd4	2.841143
19186	6370494	Psme1	2.8336074
50778	6840594	Rgs1	2.833035
	6580079	Ubp1	2.8265107
53313	2570672	Atp2a3	2.800754
	2030519	2700024H10Rik	2.799765
	2100193	1500032H18Rik	2.7934093
	4010164	2310009B15Rik	2.777243
	6270703	Pvt1	2.7744508
16859	5390131	Lgals9	2.7659416
67171	3290025	Tmem77	2.7631838
231712	4570747	Trafd1	2.7627528
170743	1570487	Tlr7	2.7226593
	1990524	4732435K05Rik	2.6945283
	540259	LOC230253	2.6830316
14252	2490739	Flot2	2.676546
	940278	Scly	2.671533
	3190468	Camk2d	2.6533422
	6960189	Furin	2.6493952
	1430035	Ralgps1	2.6412253
12802	6590196	Cnr2	2.640367
224024	3170326	Scarf2	2.6393394
	5690382	Pdk3	2.6149094
76073	4150682	0610009F02Rik	2.6102462
20937	1090328	Suv39h1	2.6051476
13040	4200646	Ctss	2.6043124
	610661	Tnfsf13b	2.601095
27215	1780681	Azi2	2.5979638
	7040202	Bcl2a1d	2.5971258

	6840059	Tnfsf13b	2.5898952
68294	4890162	0610009O03Rik	2.5799522
	3520577	0610007L01Rik	2.5767152
19157	3180196	Pscd1	2.5739574
28146	5220142	D3Ucla1	2.5719132
	2650097	4930553M18Rik	2.5712993
68758	4480368	Wbscr21	2.5701778
	2260010	Psat1	2.570035
	4590470	Ppp3cc	2.5683107
	6450369	0610009D07Rik	2.5540922
432486	3310438	Gnptab	2.5332212
15944	7550324	Irgm	2.5294955
13011	3310189	Cst7	2.528709
	6060228	1110038B12Rik	2.525459
210808	1570703	9030625A04Rik	2.5197856
26562	2760634	Ncdn	2.515578
53415	2340382	Htatip2	2.5073245
	4760189	C430026P20Rik	2.5040426
70827	730092	Trak2	2.4924257
212503	5270022	Paox	2.4877808
23853	4810543	Def6	2.4768178
19229	4200088	Ptk2b	2.4429646
	60026	Sgcb	2.4420743
108670	2760274	2310046K10Rik	2.43045
	3940129	Mela	2.4278595
93747	3450162	Echs1	2.409149
	6860307	LOC380692	2.4012818
	3360402	Max	2.3927464
	2710746	Polg	2.3817697
83704	4220112	Slc12a9	2.3665092
	6840672	U2af1-rs2	2.3641603
	2370523	Ube2h	2.3561199
	6620440	Hnrpab	2.352667
	6840598	0610039J04Rik	2.346254
	2100497	Psme2b	2.3393486
380711	1440040	Garnl4	2.3377824
	1260612	Perld1	2.3355196
	3830025	Nme2	2.3298802
	6200202	BC021438	2.3043249
	6590280	Panx1	2.2970867
66595	6270377	1100001A21Rik	2.2956433
29806	4810520	Limd1	2.2902987
	130097	Bcl2a1b	2.2734125
	4920598	Pik3ap1	2.269826
	1850500	LOC194905	2.2696452
	770403	LOC382063	2.2688954
	290300	Wrd43	2.2679827
	3850609	Jak3	2.2609313
66394	3420477	Nosip	2.2598019
66083	5090195	Setd6	2.256369
226442	2070026	Zfp281	2.2538438

	1440142	4931406P16Rik	2.2513561
	3850484	LOC208055	2.247299
103551	540398	E130012A19Rik	2.2455714
	1410646	Ube2h	2.2329316
66940	1260673	Scotin	2.2156405
	3610482	2810457M08Rik	2.2033515
	7150192	LOC228790	2.198152
	1940594	Ssh3	2.197547
	3120632	Galns	2.1928034
	2370026	Pkib	2.181093
	2710753	LOC382020	2.1746008
	3800202	LOC226574	2.1665533
17069	6550376	Ly6e	2.1643107
15466	6020594	Hrh2	2.1578815
245945	6270243	BC013481	2.1529098
100756	430754	Usp30	2.141526
66356	650164	2310008H09Rik	2.1321514
	7100608	2310032D16Rik	2.1287746
78416	430703	Rnase6	2.116824
	4040709	Gtf2f1	2.1107438
381314	6590349	2010002H18Rik	2.102973
80751	6100593	Rnf34	2.0985696
72140	3990255	2610507L03Rik	2.0932763
	2370279	Phca	2.0904293
17535	5870193	Mre11a	2.0789008
67186	240100	Rplp2	2.0750055
76938	1410131	Rbm17	2.06506
77889	5670440	Lbh	2.0650373
23986	2690717	Peci	2.0591457
67876	4830047	Coq10b	2.0422976
	2000356	Ogfrl1	2.042041
66940	6960674	Scotin	2.0203388
	610717	Ccnd3	2.0118794
	5870367	LOC268393	2.0075102
	4230019	1110007C24Rik	-2.0117767
	4230086	Slc39a13	-2.0121646
18120	5690167	Mrpl49	-2.0189633
	6510482	LOC382190	-2.0354192
	6100451	Clcn3	-2.0360396
80292	5550681	BC003332	-2.0366354
	3120431	Gtf2h2	-2.0418787
66111	4880619	Tmed3	-2.0429912
	380246	5930418K15Rik	-2.0496378
	6060370	Btbd6	-2.0536826
67057	3170370	Yaf2	-2.0640652
236920	4860445	Stard8	-2.0643702
	430551	D6Wsu176e	-2.067221
	770056	D6Wsu176e	-2.0765975
76261	4920632	0610040J01Rik	-2.0806558
23971	2650678	Papss1	-2.0832865
78323	1430142	2310046O06Rik	-2.0980737

619605	5700646	2810055E05Rik	-2.107333
	5900575	Il6st	-2.117644
20822	3840626	Trove2	-2.1202006
12972	1240025	Cryz	-2.1241736
	5270762	1700052N19Rik	-2.1262975
68520	4900484	Zfyve21	-2.1380923
68041	5130221	Mid1ip1	-2.1392205
76273	2940196	Ndfip2	-2.1429486
57376	6100577	Smarce1	-2.1507065
	4920487	Ckb	-2.151927
30928	4780609	Zfp238	-2.159984
20648	4860114	Snta1	-2.1635106
	1660397	Acvr1	-2.164739
11793	4810064	Atg5	-2.1695673
69780	1400523	1810031K02Rik	-2.1757936
228061	7320270	Agps	-2.175877
72519	5310605	2610319K07Rik	-2.18461
	3710315	Leprel2	-2.203283
	4810187	Leftb	-2.2098827
	4060176	A930033H14Rik	-2.2118201
	5340551	B130017I01Rik	-2.2139506
	6940086	Lman2l	-2.2167177
14272	7210564	Fnta	-2.2289703
380967	3850064	D15Ertd405e	-2.230131
18515	6060646	Pbx2	-2.232681
	2850192	Ppp1r2	-2.2334585
68427	6860300	Slc39a13	-2.2340014
14870	1510035	Gstp1	-2.2349076
236920	1400601	Stard8	-2.2458577
71923	1980113	2310047M10Rik	-2.2475607
	2570082	5730552M22Rik	-2.2484593
	4210152	2810025M15Rik	-2.2563262
67839	5670360	Gpsm1	-2.2617304
	6900228	scl000392.1_7	-2.2661433
23999	6520739	Ptk9l	-2.2671437
73274	2230296	Gpbp1	-2.269184
15387	6200673	Hnrpk	-2.2700613
	3290202	1110008P08Rik	-2.272455
72057	1440735	Phf10	-2.273331
12359	4760356	Cat	-2.2858098
76687	3780717	Spcs3	-2.2870016
20430	1170129	Cyfip1	-2.2876
55978	3940575	Ift20	-2.2899835
28081	5080372	D11Wsu99e	-2.2933621
17828	1110095	Muted	-2.2944536
211255	1850019	Kbtbd7	-2.3039458
76267	610274	Fads1	-2.3109221
	2810739	Zfp148	-2.313825
192654	3170577	Lypla3	-2.3195877
71954	5360064	2400003N08Rik	-2.3287828
	5130315	Cltc	-2.3421144

57376	60563	Smarce1	-2.3481042
14088	4610484	Fancc	-2.3538246
78521	3850685	B230219D22Rik	-2.3611653
	4040017	Evi5	-2.3726807
	2760241	Prkab1	-2.378881
	2650215	4631434O19Rik	-2.3805685
	5220528	D930042A21Rik	-2.3824012
20947	4730392	Swap70	-2.393863
	3610438	4933426L22Rik	-2.4030085
	2900594	2810405I11Rik	-2.4036658
18624	3400551	Pep4	-2.4122674
15473	6180445	Hrsp12	-2.4244273
66469	3460270	2810405K02Rik	-2.4252157
78689	6840300	Mak10	-2.4264145
73172	7050523	3110037I16Rik	-2.4313061
23971	520243	Papss1	-2.4329743
68910	150241	Zfp467	-2.4463012
78255	4390128	Ralgps2	-2.4471638
	5870253	Calm2	-2.4503381
69780	2470224	1810031K02Rik	-2.4525697
	3890414	2700059D21Rik	-2.4542077
140580	6450021	Elmo1	-2.4616797
	1340079	A530090C09Rik	-2.4688375
	6480450	St13	-2.4777985
214987	1990008	5830457O10Rik	-2.4842792
	4490465	Osgep	-2.487559
66105	1190504	Ube2d3	-2.48968
52666	290348	D10Ertd610e	-2.4947395
	7200360	Wdr18	-2.499602
	2360521	AA792894	-2.5012798
19645	10438	Rb1	-2.5042822
	2070152	Timp2	-2.510775
	3370068	Apg5l	-2.5122473
55980	60138	Impa1	-2.5291264
27045	4560739	Nit1	-2.5314307
	2100598	Btbd1	-2.5404923
67863	7400521	Slc25a11	-2.5526302
	3180368	Ptpns1	-2.5536509
74776	1450671	Ppa2	-2.5555077
	5910239	Eng	-2.5858016
	630327	A930010I20Rik	-2.5868418
	5340706	BC030863	-2.5897744
	4120692	Fahd1	-2.5931497
	780524	Stx1a	-2.5972373
56550	6060451	Ube2d2	-2.6011972
84094	3290239	Plvap	-2.6094868
	5050136	2610528A11Rik	-2.611304
17126	4050274	Smad2	-2.6146965
	7380291	Numb	-2.6151164
74776	4560220	Ppa2	-2.6270165
	5490524	2010005O13Rik	-2.6374931

	6400377	2310005E10Rik	-2.6377313
67608	5720564	4430402O11Rik	-2.6542733
66904	3390020	Pccb	-2.6611152
71735	4230470	1200011O22Rik	-2.66348
	5270743	Kctd12	-2.6676826
70031	7330070	Cmtm8	-2.6703935
	4150133	Pdgfa	-2.670513
241732	1090605	Tspyl3	-2.6713245
	4280041	Wrb	-2.6732972
68151	7000465	Gpr177	-2.6764617
	650291	Calr	-2.678618
71956	4490600	Rnf135	-2.6786501
74718	7040706	Snx16	-2.6896698
66308	6130255	2810021B07Rik	-2.6928527
	240202	D930009C14Rik	-2.6941142
66400	870598	2310045B01Rik	-2.6964672
66637	2450427	5730449L18Rik	-2.703721
76467	5960341	Msrb2	-2.7113369
	6290309	scl0003092.1_40	-2.7136233
	5550497	Csnk1a1	-2.7228372
23821	580446	Bace1	-2.7345595
27784	4200129	Commd8	-2.7348716
13733	2710110	Emr1	-2.7561786
14272	5960133	Fnta	-2.7586434
	6650037	Mic2l1	-2.7588727
246710	6280170	Rhobtb2	-2.7637205
66902	5080594	Mtap	-2.769087
23954	4540523	Nek3	-2.772054
	4610333	Gcn1l1	-2.7727063
	7560112	Zfp265	-2.7745771
27055	5960070	Fkbp9	-2.7746134
52004	4180270	D19Ertd144e	-2.7884989
269023	7160025	Zfp608	-2.792831
12569	7210458	Cdk5r1	-2.7932775
	1940041	Kpna3	-2.8001654
11409	270673	Acads	-2.8007205
64704	2850561	Prss25	-2.803913
	2570008	Ivns1abp	-2.813368
67213	6380044	Cklfsf6	-2.8320684
20444	5340056	Siat5	-2.8363976
12499	2320292	Entpd5	-2.8618767
13650	3840598	Rhbdf1	-2.8634522
72475	1820546	Ssbp3	-2.8683574
245522	6520474	AK129302	-2.8742294
108735	6480066	Sft2d2	-2.8751423
	5220382	B430210E12Rik	-2.88001
	130564	Rab6	-2.8820267
	3390546	Hnrph1	-2.886617
27388	630017	Ptdss2	-2.8899515
56506	6840634	Cib2	-2.9111722
19280	6220692	Ptprs	-2.911873

66530	1780168	Ubxd1	-2.914949
12361	4890246	Cask	-2.9202726
209462	3400605	Hace1	-2.930056
72027	2470086	Slc39a4	-2.9422743
71041	20025	Rnf134	-2.9442163
71780	4670634	Isyna1	-2.9450667
	5720612	0610039N19Rik	-2.9501693
76263	5270377	Gstk1	-2.9698012
109232	5340288	C330023F11Rik	-2.9774535
70031	3830427	Cmtm8	-2.9799068
	4250746	Ankrd28	-2.981395
	780746	A630054L15Rik	-2.987654
27360	5310091	Add3	-2.9970613
	630470	Mrpl35	-3.0126646
	160484	Sv2a	-3.0157974
66569	4280274	2610020H15Rik	-3.0198174
56530	7320301	Tmem4	-3.0233033
	6590309	Ube2a	-3.0296497
240168	510010	Rasgrp3	-3.0461528
	3190681	Sv2a	-3.0527527
78689	20112	Mak10	-3.0559282
26363	1510521	Btd	-3.071502
	6510100	E330027P06Rik	-3.08145
	1940215	2700046G09Rik	-3.0843163
53861	5080259	Zranb2	-3.0993726
	4290634	Rapgef5	-3.1028523
12577	4570451	Cdkn1c	-3.107184
213391	730138	Rassf4	-3.1087675
	3400546	Slc12a2	-3.1105607
	6860309	B230104P22Rik	-3.118421
	870079	E230027K01Rik	-3.1235106
140580	3450678	Elmo1	-3.1268098
	4830403	2210408O09Rik	-3.130469
21402	6900541	Skp1a	-3.13445
23885	1010521	Gmcl1	-3.1387613
13046	5090400	Cugbp1	-3.1415992
52668	160437	D12Ertd647e	-3.148253
	6400446	AV028368	-3.1506517
252972	2850154	Tpcn1	-3.151579
	7100653	7420700D11Rik	-3.1547096
	6370379	Hexa	-3.1561372
106861	150255	Abhd3	-3.1570005
30794	2940615	Pdlim4	-3.168153
	6560167	Numb	-3.169945
234776	2510376	BC060631	-3.1972623
13476	730541	Dp1	-3.1981936
	6250273	Als2cr2	-3.1985087
	4040646	Siat9	-3.2029321
	290017	9.33E+22	-3.2035012
20383	1340040	Sfrs3	-3.2036316
	6130142	E330011I20Rik	-3.2205112

	1980129	scl0001356.1_58	-3.2223022
	1230440	Mef2c	-3.2391024
	2680253	1300007O09Rik	-3.2432034
11737	1090142	Anp32a	-3.2469265
330050	1780615	AI847670	-3.248781
67416	6200630	Armcx2	-3.250545
71703	4570168	Armcx3	-3.2513294
	6660750	Fbxl19	-3.2628348
18222	4780022	Numb	-3.2956693
	6420750	Sall2	-3.3100755
70465	1990735	Wdr77	-3.3255696
13733	1030411	Emr1	-3.3382907
195522	6660626	Zfp691	-3.3391292
52668	1110280	D12Ertd647e	-3.345007
54486	5390768	Ptgds2	-3.3513024
	4830142	Rapgef5	-3.3555899
22325	4210333	Vav2	-3.366038
	5220615	Abl1	-3.3906817
102545	460347	Cmtm7	-3.3966706
66355	670626	Gmpr	-3.4017186
	2260553	Rapgef5	-3.4050438
14388	7200678	Gab1	-3.4175606
	1190619	Man2b2	-3.4205914
	2690451	LOC384318	-3.4400938
23945	2350451	Mgll	-3.4416885
22129	540202	Ttc3	-3.4477236
	5080528	1600010D10Rik	-3.457523
72759	6620195	2810439K08Rik	-3.4591973
22129	580379	Ttc3	-3.4738548
	360709	4933428I03Rik	-3.4833777
52163	7100014	Camk1	-3.489203
	1780156	Maged1	-3.4995856
	2190093	2010005O13Rik	-3.50607
56367	1850072	Scoc	-3.5504503
140580	4180403	Elmo1	-3.554041
	2680243	Gpr125	-3.5548577
214987	7200041	5830457O10Rik	-3.5779116
73847	1090228	5430432M24Rik	-3.5780027
106952	6020255	E030006K04Rik	-3.5825481
	20400	B230339H12Rik	-3.5993583
	3780369	B930007L02Rik	-3.5994353
13046	5080132	Cugbp1	-3.6020002
	3400747	Igfbp4	-3.6094816
	2320390	B430201A12Rik	-3.611147
	4010544	Slc35e3	-3.6221392
	3120706	Bin1	-3.6422768
	50152	Fbxo30	-3.6539931
	2650300	Gfer	-3.6877446
23885	3870300	Gmcl1	-3.7048757
	2120356	Myl2	-3.7100601
217364	6980647	D230014K01Rik	-3.7123995

105193	1710736	Nhlrc1	-3.7141573
	6130040	Ppp1r12a	-3.7337284
67222	5560139	Srfbp1	-3.7413044
66953	6510500	Cdca7	-3.755155
	6250593	1110064N10Rik	-3.7691076
233274	70463	Siglech	-3.7765267
	7400632	Fbxl19	-3.7860851
56534	2940243	Hspb3	-3.808328
66922	4230142	Rras2	-3.811914
	7560170	A930029N17Rik	-3.8226185
	4610653	1700081H05Rik	-3.8229976
	6400017	Hist1h2bc	-3.8586414
60365	1710152	Rbm8	-3.878243
21812	3940184	Tgfbr1	-3.8912659
212276	2320187	Zfp748	-3.9107764
	1240296	Clstn1	-3.9163566
216881	6250082	BC030477	-3.922851
67266	60601	2900024C23Rik	-3.928497
70178	50608	2210412D01Rik	-3.9330773
26365	1050465	Ceacam1	-3.9466677
	1230243	D030028A08Rik	-3.9510322
102545	1980768	Cmtm7	-3.9643934
	6270270	Spg20	-3.9763305
	5890059	Epb4.9	-3.9767656
20621	1030577	Snn	-3.9974792
	990634	1700129L13Rik	-4.0077596
	5890097	Sema4b	-4.0536036
	6580100	Spg20	-4.0544896
207521	1300300	Dtx4	-4.070011
	610377	C430010P07Rik	-4.076873
60365	4900040	Rbm8	-4.083848
	7510692	C630025L14	-4.090974
	3460670	Oxct1	-4.0915565
56468	270678	Socs5	-4.126411
	4070025	Slc12a2	-4.1331034
102545	6380326	Cmtm7	-4.1434383
54632	130431	Ftsj	-4.1470785
	4850747	Eif4a2	-4.164081
140580	3130600	Elmo1	-4.1640854
	1170709	D13Bwg1146e	-4.164168
	1010110	Rab11a	-4.1652327
	4830220	Epn2	-4.1706996
235584	4540056	Dusp7	-4.1761136
212627	6770386	Prpsap2	-4.223417
331474	360608	Rgag4	-4.229003
23945	5310328	Mgll	-4.2589927
	5690156	Pes1	-4.2597136
320736	5420445	E130203B14Rik	-4.2615814
207521	130102	Dtx4	-4.2827873
17179	3440397	Matk	-4.2866797
78408	4920079	2900046G09Rik	-4.3402166

	150707	9530051E16Rik	-4.355264
	5870044	1110012N22Rik	-4.3558745
20363	380133	Sepp1	-4.3608804
16430	1410196	Itm1	-4.3746743
	4040632	Ube2n	-4.4141397
76416	6280270	1700022C21Rik	-4.423207
329165	70746	Abi2	-4.446644
73710	1030133	Tubb2b	-4.4905324
30948	290242	Bin1	-4.50301
329165	6060441	Abi2	-4.5087266
	5860608	Rbbp4	-4.5126247
	1990138	C130085G02Rik	-4.5133095
	3930093	LOC232532	-4.5423408
	2260280	Epn2	-4.5589848
	3310368	Endogl1	-4.5623093
	4830709	scl0002124.1_39	-4.5625286
60322	4640278	Chst7	-4.5738378
72324	7570619	Plxdc1	-4.5834675
	5560711	1200006F02Rik	-4.604957
109711	4590136	Actn1	-4.625583
140580	5220554	Elmo1	-4.6484623
	6770450	Slc37a1	-4.688916
	2490575	Serpinf1	-4.6989546
59030	7200068	Mkks	-4.7009463
21349	1580035	Tal1	-4.7079153
18074	6370035	Nid2	-4.7721443
	3120403	F9	-4.7821255
58245	5260082	Gpr180	-4.8360868
70237	460239	Bhlhb9	-4.846374
	2450343	Hpgd	-4.854795
	2940064	LOC384853	-4.8811364
	1710300	5730590C14Rik	-4.9243293
	2630204	Lbx2h	-4.9323206
13733	7610372	Emr1	-4.937399
70839	1440050	P2ry12	-4.956196
	3710209	A2bp1	-4.9656987
	1110048	Unc13b	-4.9703794
	6330086	Leprel1	-4.977682
	7650273	Hist1h1c	-4.977993
12745	6980487	Clgn	-5.025053
223332	4610131	C130037N17Rik	-5.031054
319625	5720022	Galm	-5.0535674
	6180632	Fts	-5.0552464
99326	4610288	Garnl3	-5.1180186
	1090671	Inpp4b	-5.166094
68092	1230615	Ncbp2	-5.19578
319845	160291	E130103I17Rik	-5.2187443
66870	3370010	Serbp1	-5.2325816
	1090397	LOC380658	-5.2683644
56807	7160372	Scamp5	-5.32189
	6100520	Cnnm2	-5.323761

	3830524	Magee1	-5.364121
	2450450	LOC240261	-5.3735914
	20615	Ppm1l	-5.3815427
	5050066	Ppm1l	-5.4096684
70652	20706	5730537D05Rik	-5.433914
	4810328	Nrarp	-5.4685273
52637	110576	D10Ertd214e	-5.472129
	450370	4921509E05Rik	-5.592003
93872	4010709	Pcdhb1	-5.628004
14697	6760750	Gnb5	-5.689111
	2340670	4921506I22Rik	-5.7023134
	5570132	Ppm1l	-5.739198
56807	3850280	Scamp5	-5.7585073
	3180300	4930404K22Rik	-5.7709937
	3710750	BC027174	-5.8164263
	5860338	1700008D07Rik	-5.882566
	1110035	Rab3ip	-5.9651823
	5690092	Ak1	-6.0053205
	1340619	Ly6g6d	-6.0373063
80884	360437	Maged2	-6.0998144
72865	4730168	Cxx1c	-6.1466784
13803	6290689	Enc1	-6.2557416
71827	4250239	Lrrc34	-6.375852
83679	6100612	Pde4dip	-6.3914156
207278	5260079	Fchsd2	-6.4057755
18451	5870040	P4ha1	-6.4596953
55936	4060291	Ctps2	-6.559136
20621	4860373	Snn	-6.7751412
	20739	2310040G24Rik	-6.8034964
320705	6290403	B230209C24Rik	-6.8057814
20363	7160603	Sepp1	-6.8085084
13641	1400152	Efnb1	-6.829031
	3870392	Bglap1	-6.8421664
80891	6130746	Msr2	-6.8579535
654812	1980605	Angptl7	-6.869553
	3940193	LOC238678	-6.8943324
18074	5960066	Nid2	-6.9113126
56504	20465	Stk23	-6.9174123
	670440	Rgs2	-7.050744
	1570022	Bcl11a	-7.165982
67374	1470215	Jam2	-7.182305
	3460021	Abcf3	-7.214351
	1690255	1700017I11Rik	-7.242002
	7210373	A630002K24	-7.3159823
	5900356	scl000028.1_0_REVCOMP	-7.346039
12153	6940278	Bmp1	-7.4499125
66082	6200240	Abhd6	-7.4518394
	2190161	1700110I01Rik	-7.5083895
53951	6590189	2310002B06Rik	-7.625136
	4540221	Fscn1	-7.631894

23945	6660719	Mgll	-7.6540003
320333	1580386	D830030K20Rik	-7.688714
	7330445	LOC381755	-7.7194457
	5270195	P4ha1	-7.729238
	1410634	Gtf2h2	-7.797749
	290561	scl0002702.1_3805	-7.8292947
22696	2000307	Zfp37	-7.8382883
	1850315	Sox4	-8.068424
67425	7400707	Eps8l1	-8.17454
225027	5700541	Sfrs7	-8.2021475
27528	7040243	D0H4S114	-8.293479
	270601	1700110I01Rik	-8.297816
	450091	Msr2	-8.450049
74211	7560201	1700017B05Rik	-8.622992
	6650477	Ian6	-8.728201
	1940040	4931406O17Rik	-8.931486
	2630364	1110021L09Rik	-9.062013
68947	4590113	Chst8	-9.073578
52331	1430129	D5Ertd593e	-9.181482
	830736	Adra2a	-9.278435
	7570162	scl0001297.1_42	-9.2978735
	4810132	Lrba	-9.499885
	3120129	Gprasp1	-9.513486
	3140369	LOC228238	-9.56494
230766	3120402	BC030183	-9.658654
	110465	1700094J05Rik	-9.668645
	5570519	Usp48	-9.808927
	580259	Catnd2	-9.864736
330217	6330068	Gal3st4	-9.962367
	7150327	2610203C22Rik	-10.233364
	5810576	Cxcr3	-10.400648
72948	2140431	2900041A09Rik	-10.529157
	1410673	Ppm1e	-10.640428
	6250673	2310047D07Rik	-10.701365
	4880762	6430524C05Rik	-10.721642
117590	6400360	Asb10	-10.909695
	780035	Gprasp1	-10.910683
21812	4640750	Tgfbr1	-10.939467
	5690403	Gadl1	-10.949318
237052	4570468	Tceal1	-11.205791
66642	1820019	Ctnnbl1	-11.367433
69032	2340255	Lyzl4	-11.442951
	3990228	9330161C17Rik	-11.486881
	7560554	Jam3	-11.701891
237052	840762	Tceal1	-11.706359
76722	430427	Ckmt2	-11.811412
67247	70468	2810484M10Rik	-11.849452
18670	7210136	Abcb4	-11.868487
	7050661	6330419J24Rik	-12.172128
23890	7510333	Gpr34	-12.325673
	4200164	LOC381900	-12.328836

407831	5360750	BC054438	-12.389819
14772	650692	Gprk2l	-12.399905
117589	1440477	Asb7	-12.550074
	360484	LOC239426	-12.7721615
23890	3290240	Gpr34	-12.972253
54368	4280414	Gp9	-13.196512
70652	2490291	5730537D05Rik	-13.570747
	3170021	Tef	-13.592133
65256	2850398	Asb2	-13.979256
	4920491	6720458D17Rik	-14.01315
210741	6060242	Kcnk12	-14.159665
	6220142	Khdrbs3	-14.17978
	3450333	Mapk8ip3	-14.474054
	2630494	LOC382527	-14.649677
67441	2030474	0610042E07Rik	-15.096732
	1980746	6530406P05Rik	-15.458729
14137	3710358	Fdft1	-15.805224
	520692	E130115E03Rik	-15.991478
21835	6580403	Thrsp	-16.040825
	6420537	Aebp2	-16.571474
	2320619	Elavl4	-16.829538
	1740091	Pfkp	-17.21422
	7570390	9330175B01Rik	-17.275976
	2120576	mtDNA_ND6	-17.915466
	840148	Sncaip	-17.957117
	3180452	Zfp286	-18.37543
	670072	Hspa5bp1	-18.59753
109245	2690524	9430028I06Rik	-18.738008
	4060270	2410078J06Rik	-18.797136
13885	4010563	Esd	-21.67733
22268	4880433	Upk1b	-21.744942
	4040711	LOC380876	-22.142038
14204	6020224	Il4i1	-22.431118
	2850347	Lt1	-24.978193
	3140259	9030205A07Rik	-25.373133
241556	6180524	6720430O15	-28.405855
	3140397	Gprasp2	-31.16595
	6660202	3526401B18Rik	-32.624947
21935	2940482	Tnfrsf17	-34.03184
67374	7570673	Jam2	-34.592003
	780202	D030035F05Rik	-35.15158
214968	6510270	Sema6d	-35.219437
80877	5220333	Lrba	-36.416218
239157	7040386	Pnma2	-43.546467
	5810598	Col6a3	-52.25674
	4200546	Adcy8	-64.21477
241556	2320035	6720430O15	-65.34124
271127	3130450	Adamts16	-69.13274
	5080296	2610042G18Rik	-81.00859
16644	4290521	Kng1	-92.20496

Supplementary table S3: Upregulated genes at d5 and d7

Entrez Gene ID	ProbeID	Gene symbol	Fold change vs naïve day 5	Fold change vs naïve day 7
	1050092	Serpina3g	75.3	1178.3
	3170487	F730004D16Rik	29.9	624.3
	540647	Cd69	42.9	414.2
18175	3170047	Nrap	160.5	394.1
	4730246	LOC226690	244.6	378.6
231655	4780253	Oasl1	562.7	376.1
246728	3060450	Oas2	232.1	246.0
	7400528	Ifi205	27.7	243.7
	6450259	Ifi205	52.4	234.7
231655	240187	Oasl1	159.2	199.6
	60368	Slp	67.9	189.2
14469	6860600	Gbp2	19.0	185.8
68713	4010019	Ifitm1	66.2	181.2
	130348	Slp	66.9	175.1
	5820709	LOC240921	14.1	157.6
	10598	Cxcl9	14.7	137.2
	2190133	Ifi205	48.8	123.9
70218	2680010	3000004C01Rik	354.9	123.5
	4810184	Dlm1-pending	25.5	122.8
	580470	Tnfrsf5	40.8	122.4
20555	3520746	Slfn1	34.8	121.5
58185	5560474	2510004L01Rik	25.7	120.9
	4570458	Sn	99.4	116.4
231655	840259	Oasl1	44.3	113.6
	3840575	Cd244	21.6	113.0
	6590653	Irf7	69.8	101.7
12268	6510113	C4	40.4	93.6
	2810612	Birc5	391.3	90.8
15958	5270398	Ifit2	19.8	78.7
	2190164	Ccnb1	236.3	77.8
12268	7510243	C4	36.3	77.4
15953	1090139	Ifi47	9.2	73.7
15958	360041	Ifit2	10.6	72.5
15951	7550093	Ifi204	28.7	68.8
	6020487	Cyp4f18	13.4	68.1
	3780474	9930016I07Rik	28.0	66.9
231507	5860243	Plac8	53.2	63.8
	1500446	Cdca8	103.0	58.5
70218	1710368	3000004C01Rik	275.6	57.6
	6420170	BC006779	64.2	54.9
16571	6660050	Kif4	287.9	53.2
229003	450706	BC006779	49.1	52.5
68713	5960577	Ifitm1	30.0	51.5
	160041	Mmp25	19.2	50.1
	430202	Top2a	63.5	49.0
	2190215	Cxcl10	9.6	47.9

	1240538	P2ry14	26.3	46.2
	7400215	Birc5	169.7	44.0
	2630066	Ckap2	175.8	43.7
219131	6660327	Phf11	19.6	43.6
	3290292	Mx1	8.2	42.2
102084	520040	AI451557	17.5	42.0
219131	1340193	Phf11	20.8	40.5
15959	3460504	Ifit3	23.4	40.5
15945	3140209	Cxcl10	13.4	39.1
14469	1400041	Gbp2	10.1	37.9
19340	1470278	Rab3d	6.1	37.3
18610	3610332	Pdyn	9.0	37.0
66141	3840292	Ifitm3	17.2	34.7
	3830328	Cdc25c	81.8	34.7
12263	4210470	C2	16.7	34.5
23962	7050014	Oasl2	108.4	34.5
	3840523	Kif23	46.2	33.9
	1410554	Tyki	18.9	33.6
	6580379	P2ry14	15.1	33.4
71819	4850079	Kif23	140.2	33.4
23962	4830010	Oasl2	20.3	32.6
	1240446	Birc5	111.3	31.8
58203	1580634	Zbp1	20.9	31.2
217430	5890523	Pqlc3	4.3	31.0
	540438	LOC328752	31.4	29.6
	5820128	0610037M15Rik	24.2	28.8
15959	7510020	Ifit3	13.4	28.3
20684	1170110	Sp100	24.8	27.4
	2850717	H2-Q8	34.9	27.3
	4830543	2310061N23Rik	19.7	26.7
21822	3890121	Tgtp	31.0	26.4
	3940242	AA175286	10.4	26.3
14190	2340242	Fgl2	14.1	26.1
53606	460221	G1p2	16.1	24.6
227659	7400286	Slc2a6	6.6	24.0
228421	3180164	Kif18a	92.3	23.6
	6280521	A530060O05Rik	4.8	23.2
233406	780475	Prc1	49.9	22.8
73804	1780543	Kif2c	16.3	22.5
80876	840349	Ifitm2	7.5	22.1
	1090647	9830148G24Rik	12.4	21.1
24110	4610139	Usp18	25.0	20.8
	2490653	LOC240327	15.7	20.4
	7040475	2610019I03Rik	121.1	20.1
67775	2760577	Rtp4	14.0	20.0
59027	2750189	Pbef1	11.0	19.8
21853	160703	Timeless	30.2	19.3
	2230538	Cd44	5.4	18.1
	3890682	2610036L11Rik	18.8	18.0
	2650703	9830002L24Rik	48.7	17.9

74107	3710050	1200008O12Rik	67.9	17.5
12268	1010632	C4	4.7	17.4
	2350095	Bub1b	29.0	17.3
15019	2750128	H2-Q8	26.3	17.2
24110	2490452	Usp18	16.8	16.9
327957	1190477	A430084P05Rik	3.4	16.3
14793	1050170	Cdca3	42.6	15.6
	4040095	H2-Q6	9.0	15.6
20847	6220594	Stat2	7.9	15.2
24110	1580528	Usp18	12.4	15.2
	1400220	LOC215405	10.4	14.7
23960	3890328	Oas1g	19.5	14.7
23960	130598	Oas1g	29.2	14.2
	4850689	A130019H11Rik	6.7	14.2
19200	2360554	Pstpip1	7.0	14.0
60533	3870561	Pdcd1lg1	2.0	13.7
107995	4610722	Cdc20	25.5	13.6
	1090382	Cdkn3	18.9	13.4
110454	1690187	Ly6a	17.3	13.1
74747	130634	Ddit4	14.5	13.0
	6450634	1110017L21Rik	49.2	12.9
70466	2450270	2610318C08Rik	25.6	12.8
217946	4220139	BC006933	11.2	12.7
246256	830632	Fcrl3	4.7	12.4
	5390088	LOC327957	3.5	12.3
80861	6330592	D11Lgp2e	7.7	11.9
	520278	Mx2	5.9	11.8
	5720397	D11Lgp2e	9.0	11.7
219132	2810040	D14Ertd668e	5.6	11.2
	6450598	D11Lgp2e	9.3	11.1
547343	1400132	LOC547343	4.7	10.9
15944	5820608	Irgm	4.6	10.6
12575	2070377	Cdkn1a	4.7	10.6
21354	5870093	Tap1	4.6	10.6
	6290592	LOC226691	6.2	10.5
414801	2940612	BC063749	3.2	9.9
	1110445	LOC380706	6.4	9.8
20556	4120307	Slfn2	5.8	9.7
12575	7040491	Cdkn1a	4.1	9.6
	6760762	Sdc3	5.9	9.3
12575	3130630	Cdkn1a	3.4	9.1
667977	270367	H2-gs17	6.5	8.9
219132	520053	D14Ertd668e	4.5	8.9
22021	2120463	Tpst1	4.3	8.7
	3140446	2310020F24Rik	3.5	8.6
56045	3190577	Samhd1	2.3	8.3
26362	7100577	Axl	3.6	8.3
12257	6270709	Tspo	4.2	7.8
	160463	Lgals3bp	4.3	7.7
12257	4670544	Tspo	5.3	7.6

12257	2760544	Tspo	4.8	7.6
	7200519	Cenpa	11.7	7.6
	2510333	Ccl12	4.3	7.6
	50059	AI504432	3.5	7.6
15024	870446	H2-T10	5.5	7.1
110749	7200270	Chaf1b	12.7	6.9
56628	1710768	LOC56628	5.5	6.6
	3400491	Clic4	2.8	6.6
	990615	LOC239122	3.2	6.6
16362	3360138	Irf1	2.1	6.4
14972	6280026	H2-K1	4.6	6.4
	6350114	Ube2l6	8.3	6.3
	6280221	Sp100	7.8	6.3
	5270475	Trex1	3.4	6.1
	6590215	H2-T22	4.1	6.1
	1990221	Ccl12	2.7	6.0
630499	4640348	EG630499	8.3	5.9
	1470605	D11Ertd759e	4.6	5.7
	6350189	Trex1	3.3	5.6
55932	60553	Gbp4	2.8	5.6
16319	630634	Incenp	7.8	5.5
	2650326	Pml	3.8	5.4
	2970598	Map3k8	2.3	5.3
	6450682	H2-L	3.3	5.3
	6250600	Cdkn2d	4.7	5.3
55932	1690475	Gbp4	3.4	5.2
74137	1770592	1200013B22Rik	2.6	5.2
	830537	LOC269515	5.2	5.1
15018	6940386	H2-Q7	3.9	4.9
	6760390	H2-L	3.0	4.6
15013	5720048	H2-Q2	3.6	4.6
	380215	Tor3a	2.1	4.5
56417	1780154	Adar	2.9	4.4
	2650754	Ube2l6	8.1	4.4
	780221	H2-T9	3.3	4.3
243771	1340050	Zc3hdc1	3.1	4.3
14964	4060735	H2-D1	2.1	4.2
	1780452	Fbxw17	3.0	4.2
	4490239	AI481105	2.2	4.2
	2230731	H2-T17	3.4	4.2
15040	4010154	H2-T23	2.6	4.1
	3780736	H2-K1	3.3	4.0
	10167	LOC56628	2.6	4.0
380732	5960678	Gm885	2.8	4.0
15040	7200100	H2-T23	2.6	3.9
	4120014	Ifi35	2.8	3.8
547253	2970521	Parp14	3.4	3.7
	2490142	Ddx58	3.2	3.7
	4730367	Ifi30	3.2	3.4
12363	5290017	Casp4	2.0	3.3

	4290709	Ifi30	2.8	3.3
	1940338	H2-M3	2.6	3.2
12517	5700528	Cd72	6.3	3.2
	10402	Trim25	2.0	3.2
	3460037	Fen1	3.9	3.0
76073	4150682	0610009F02Rik	2.2	2.6
108670	2760274	2310046K10Rik	2.9	2.4
	4920598	Pik3ap1	2.1	2.3
17069	6550376	Ly6e	2.5	2.2
72140	3990255	2610507L03Rik	2.8	2.1

Supplementary table S4: Genes related to type I IFN signal

Entrez Gene ID	ProbeID	Gene symbol	Fold change infected vs naïve day5	Fold change infected vs naïve day7
	540647	CD69	42.887672	414.19824
18175	3170047	NRAP	160.49538	394.08554
231655	4780253	Oasl1	562.70	376.06052
53608	7200025	Map3k6	<2	289.86234
246728	3060450	Oas2	232.05	246.03413
	7400528	Ifi205	52.38	243.71439
	7560307	IRG1	<2	205.81625
	5340577	XDH	<2	191.93933
14469	6860600	Gbp2	18.95	185.7916
68713	4010019	Ifitm1	66.18	181.21935
58217	4280504	TREM1	<2	141.02757
	10598	Cxcl10	9.64	137.17833
20555	3520746	Slfn1	34.845673	121.54738
	6590653	Irf7	69.78	101.734886
	2810612	BIRC5	391.27356	90.84561
18712	5860039	PIM1	<2	82.135574
15951	3060554	Ifi204	28.72	79.68101
15958	5270398	Ifit2	19.83	78.73005
15953	1090139	Ifi47	9.20	73.65926
231507	5860243	PLAC8	53.18062	63.796135
16169	6400270	IL15RA	<2	57.363308
15959	670670	Ifit3	23.40	48.86549
	4890689	oas3	<2	48.21454
	2190215	Ctla2b	<2	47.90023
	3290292	Mx2	5.88	42.183247
14967	5050451	H2-D4	<2	39.440598
16174	430446	IL18RAP	<2	38.17255
57444	540411	ISG20	<2	35.519783
66141	3840292	Ifitm3	17.21	34.70865
23962	7050014	Oasl2	108.35	34.468334
58203	1580634	ZBP1	20.905413	31.169638
20304	1690768	Ccl5	<2	30.106688
20684	1170110	Sp100	24.784704	27.380056
	2850717	H2-Q8	26.31	27.256311
21822	3890121	Tgtp	30.97	26.397688
14190	2340242	FGL2	14.135186	26.0683
	3420433	IL12RB1	<2	22.17348
80876	840349	Ifitm2	7.52	22.117163
20307	2480296	Ccl8	<2	21.234295
24110	4610139	Usp18	25.03	20.82286

67775	2760577	RTP4	14.017168	20.039913
20847	6220594	Stat2	7.85	15.200038
23960	3890328	Oas1g	29.22	14.694639
23961	5550010	Oas1b	<2	14.200897
110454	1690187	Ly6a	17.34583	13.109668
74747	130634	DAXX	<2	13.0422535
	270193	TNFSF13B	<2	12.3504
	520278	Mxd3	31.19	11.761493
12575	2070377	CDKN1A	4.6513176	10.639942
21354	5870093	TAP1	4.6280155	10.6270895
15016	6980075	H2-Q5	<2	9.767257
	2490328	PML	3.967862	9.696
20556	4120307	Slfn2	5.795018	9.694468
667977	270367	H2-gs17	6.54	8.926894
22021	2120463	TPST1	4.3107605	8.743125
	3290707	TNFSF10	<2	8.595983
21816	6270040	TGM1	<2	8.562749
56045	3190577	SAMHD1	2.2548714	8.289592
26362	7100577	AXL	3.6006958	8.283421
	160463	LGALS3BP	4.325593	7.654424
	2510333	Ccl12	4.25	7.570478
15024	870446	H2-T10	5.49	7.133779
16362	3360138	Irf1	2.05	6.442806
14972	6280026	H2-K1	3.32	6.434294
	6350114	UBE2L6	8.326761	6.2942476
	5270475	TREX1	3.4327192	6.081574
55932	60553	Gbp4	3.35	5.602284
	4150369	CXCR3	<2	5.3743515
	2970598	MAP3K8	2.3416035	5.332027
12046	3520735	BCL2A1c	<2	4.9259906
229900	6330692	Gbp6	<2	4.8516703
15018	6940386	H2-Q7	3.88	4.8510656
78781	520600	ZC3HAV1	<2	4.673763
15013	5720048	H2-Q2	3.62	4.595039
56417	1780154	ADAR	3.6127155	4.4280953
15040	2350181	H2-T23	2.60	4.328157
14964	4060735	H2-D1	2.10	4.2292175
	2940138	CLSTN1	<2	4.176888
12608	4230348	CEACAM1	<2	4.0453777
	4120014	Ifi35	2.77	3.7912788
547253	2970521	PARP14	3.3897967	3.7473128
	2490142	DDX58	3.204791	3.67007
	4730367	Ifi30	3.20	3.4139001
12363	5290017	CASP4	2.0126648	3.2787597

19186	940674	PSME1	<2	2.9852178
	5670398	TRIM21	<2	2.8592765
	1110088	APOBEC1	<2	2.8509338
170743	1570487	TLR7	<2	2.7226593
17069	6550376	LY6E	2.453925	2.1643107
	610717	CCND3	<2	2.0118794
	5900575	IL6ST	-2.3983967	-2.117644
19645	10438	RB1	<2	-2.5042822
	2070152	TIMP2	<2	-2.510775
27055	5960070	FKBP9	<2	-2.7746134
	3400747	IGFBP4	<2	-3.6094816
	1240296	CEBPB	<2	-3.9163566
26365	1050465	CDKN2D	4.655705	-3.9466677
18074	5960066	NID2	<2	-6.9113126
	5810576	Cxcl9	14.71	-10.400648
21812	4640750	TGFBR1	<2	-10.939467
14204	6020224	IL4I1	<2	-22.431118
		Ifitm6	10.41	<2
		Mx1	8.18	<2
		Stat1	5.55	<2

Supplementary table S5: Combination of primers and probes for qRT-PCR.

Gene symbol	Forward primer	Reverse primer	Probe
**HPRT**	tcctcctcagaccgctttt	cctggttcatcatcgctaat	95
**Oasl1**	ggccaaccagtgtctgaaa	tggatatcgggtgctctctt	78
**IRF7**	cttcagcactttcttccgaga	tgtagtgtggtgacccttgc	25
**H2Q8**	cctgaggactgcacagagc	ggccttcataggcgaactg	76
**Cxcl10**	gctgccgtcattttctgc	tctcactggcccgtcatc	3
**Ccl5**	tgcagaggactctgagaca	Gagtggtgtccgagccata	110

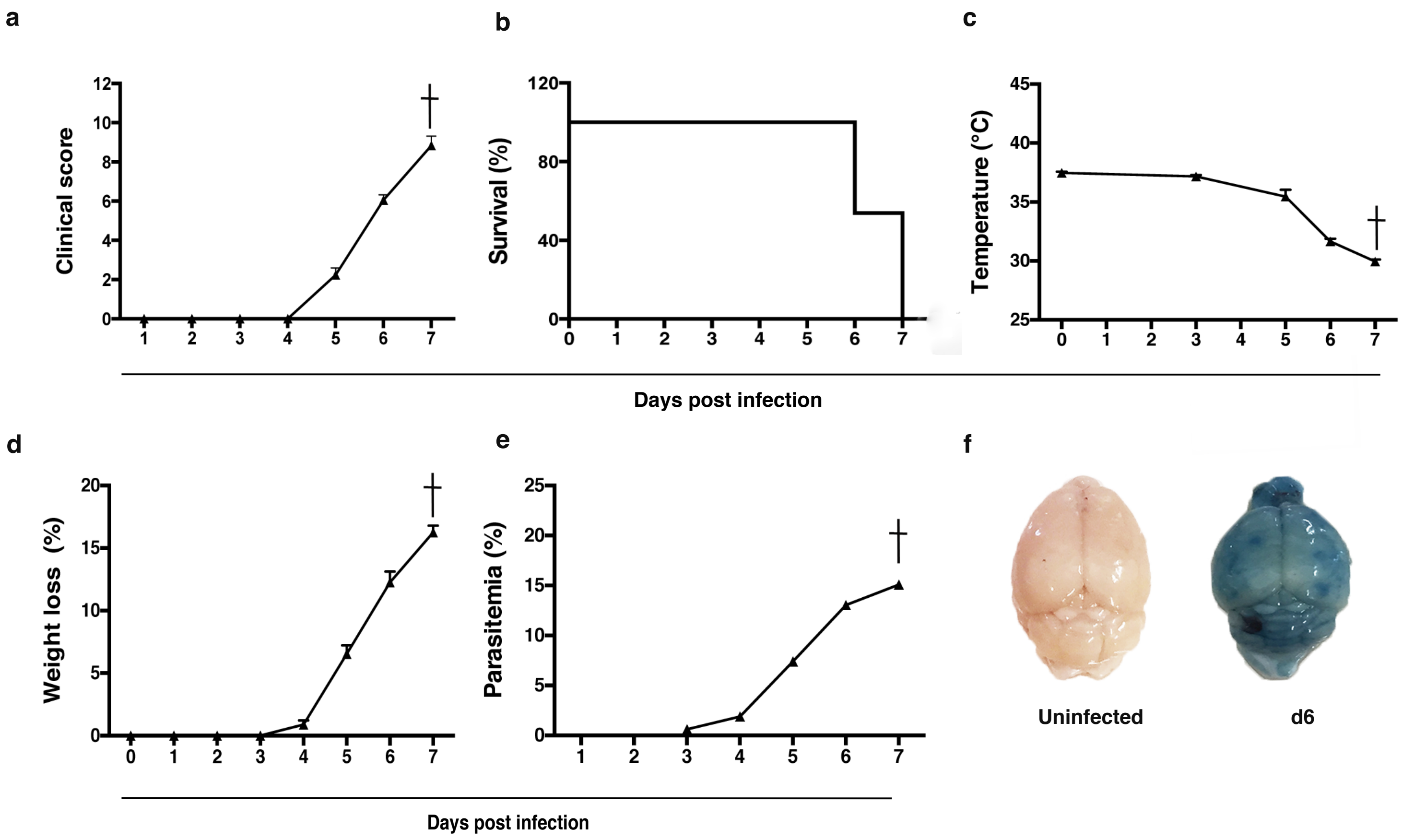

Figure S1. Experimental Cerebral Malaria in C57BL/6 mice infected with Plasmodium berghei ANKA. C57BL/6 mice were infected with 10^5 PbA infected RBCs injected ip. (a) Clinical score of the mice was calculated as the sum of several neurological signs and phenotypic features as described in the materials and methods; (b) Mortality (mice reached humane endpoint when their temperature was below 31ºC); (c) body temperature; (d) body weight; (e) parasitemia as percentage of infected red blood cells (F) Representative images of Evans blue dye extrusion analysis from PbA infected mice at day 6 post-infection. Results in graphs A, C and D are represented as mean ± SEM, n = 9 from 3 separate experiments. The results in graph E are represented as geometric mean.

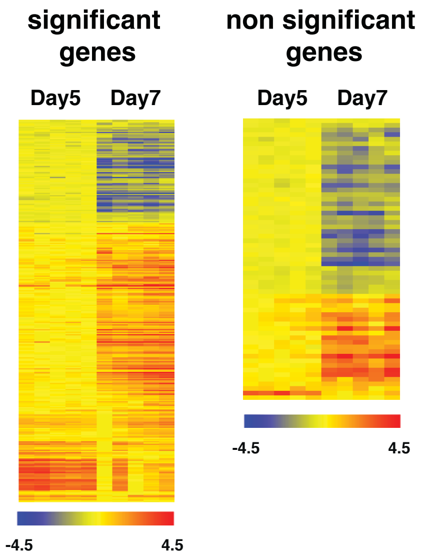

Figure S2
Heatmaps representing genes differentially expressed at both, d5 and d7.
On the left heatmap are clustered all the genes that passed the statistical analysis ($p<0.05$, FC>2), while on the right one are clustered genes that didn't pass the statistical analysis ($p>0.05$, FC>2).

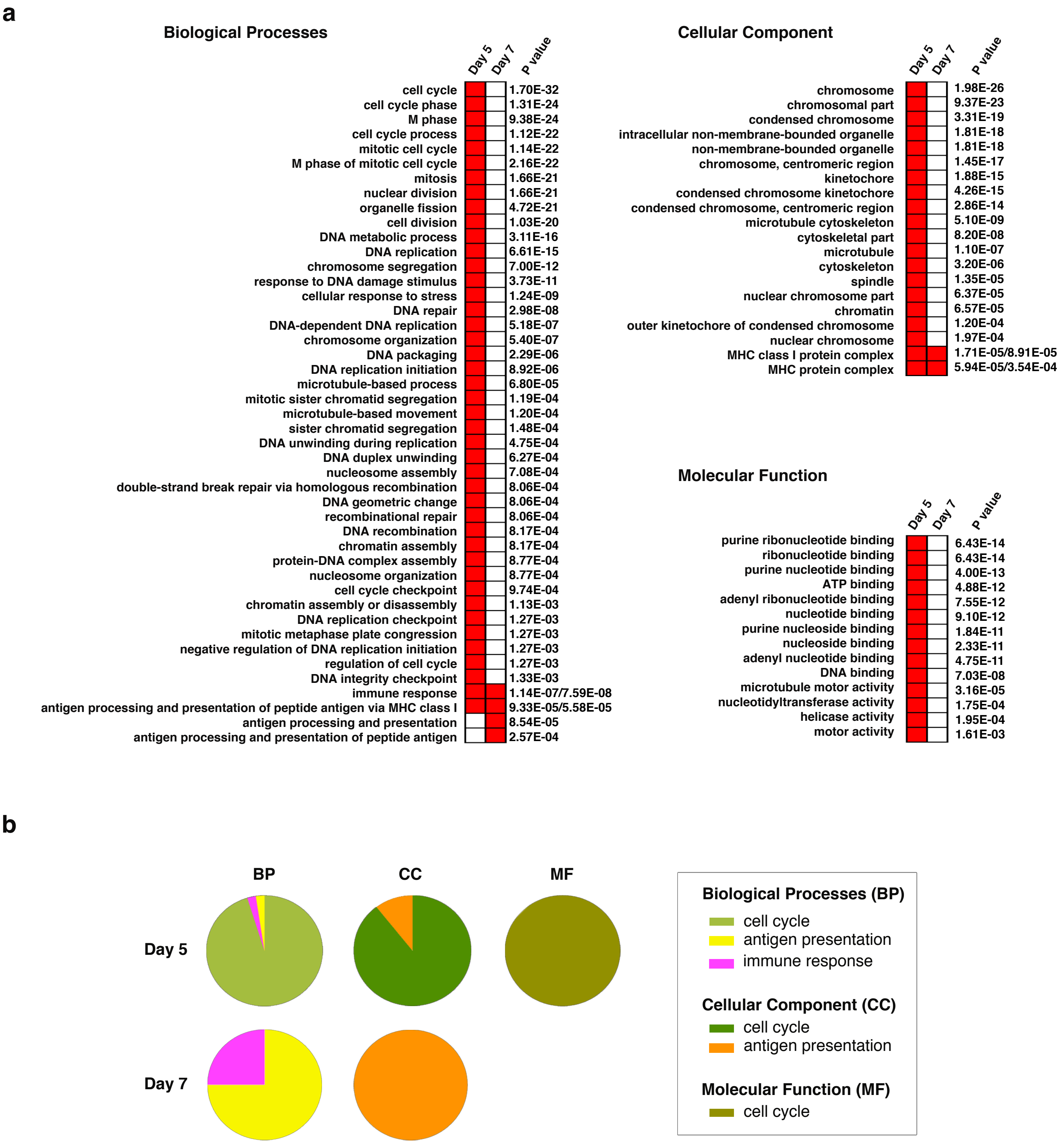

Figure S3
Gene Ontogeny (GO) analysis of differentially expressed genes in microglia during ECM.
(a) Enriched terms (red colour) in Biological processes, Cellular Component and Molecular function using DAVID ($p<0.001$) are listed and compared between day 5 and day 7 of infection with PbA. p values for each enriched terms are reported on the side. (b) Pie charts representing the distribution of the significant GO terms enriched in biological processes (BP) cellular component (CC) and molecular function (MF) at day 5 and day 7.

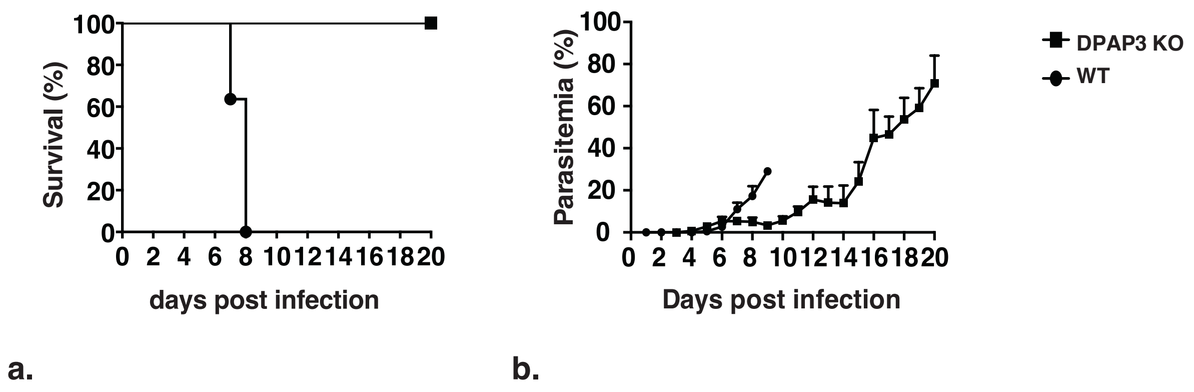

Figure S4

C57BL/6 mice were infected with 10^4 DPAP3KO iRBC. Survival (a) and parasitemia (b) were monitored daily from d3. Results are represented as mean ± SD, n=10.

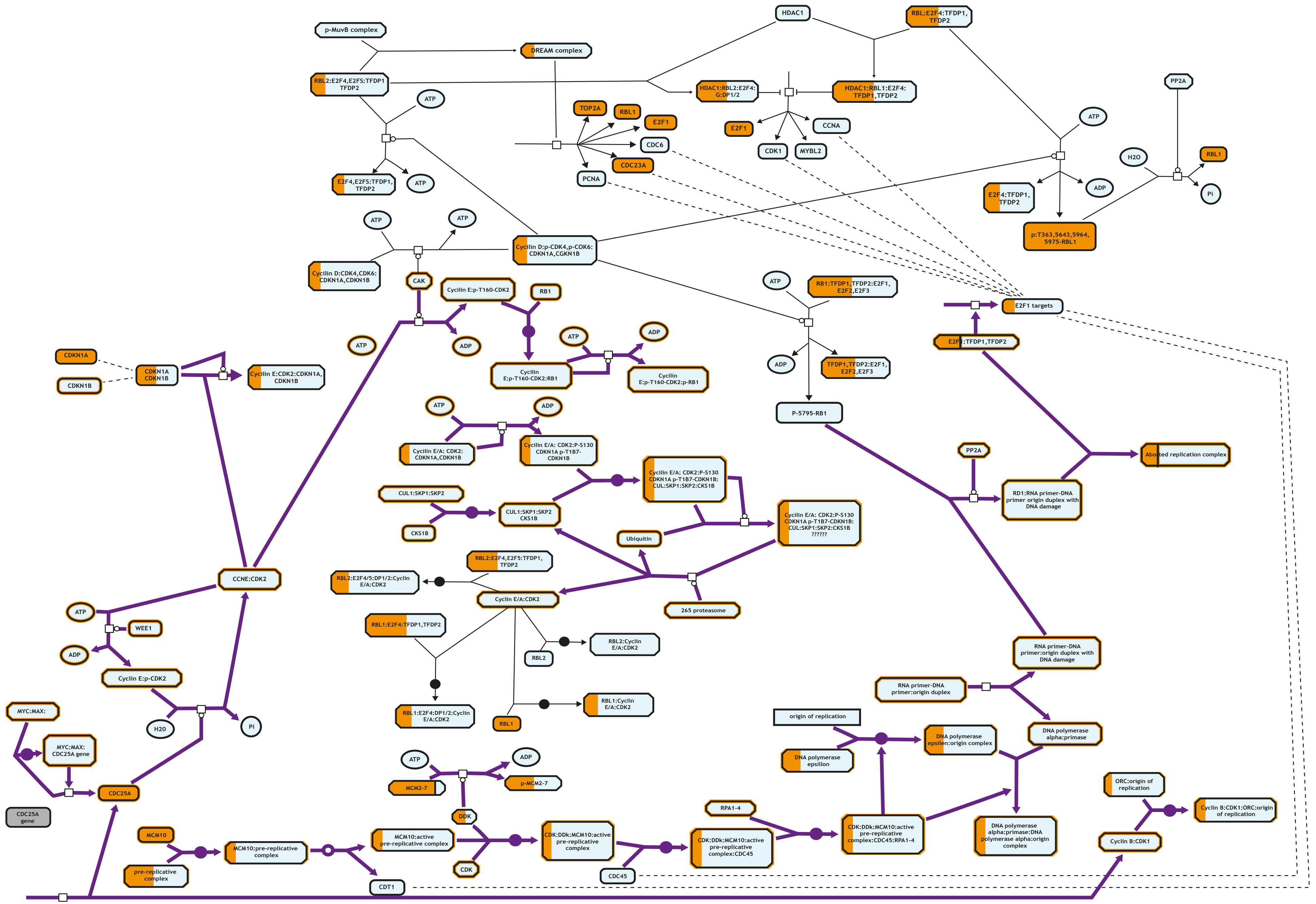

Figure S5.
Representation of "Cell Cycle" pathway. In orange are represented all the DE transcripts at d5. Boxes containing more genes have a partial orange coloration depending on the content of DE transcripts.

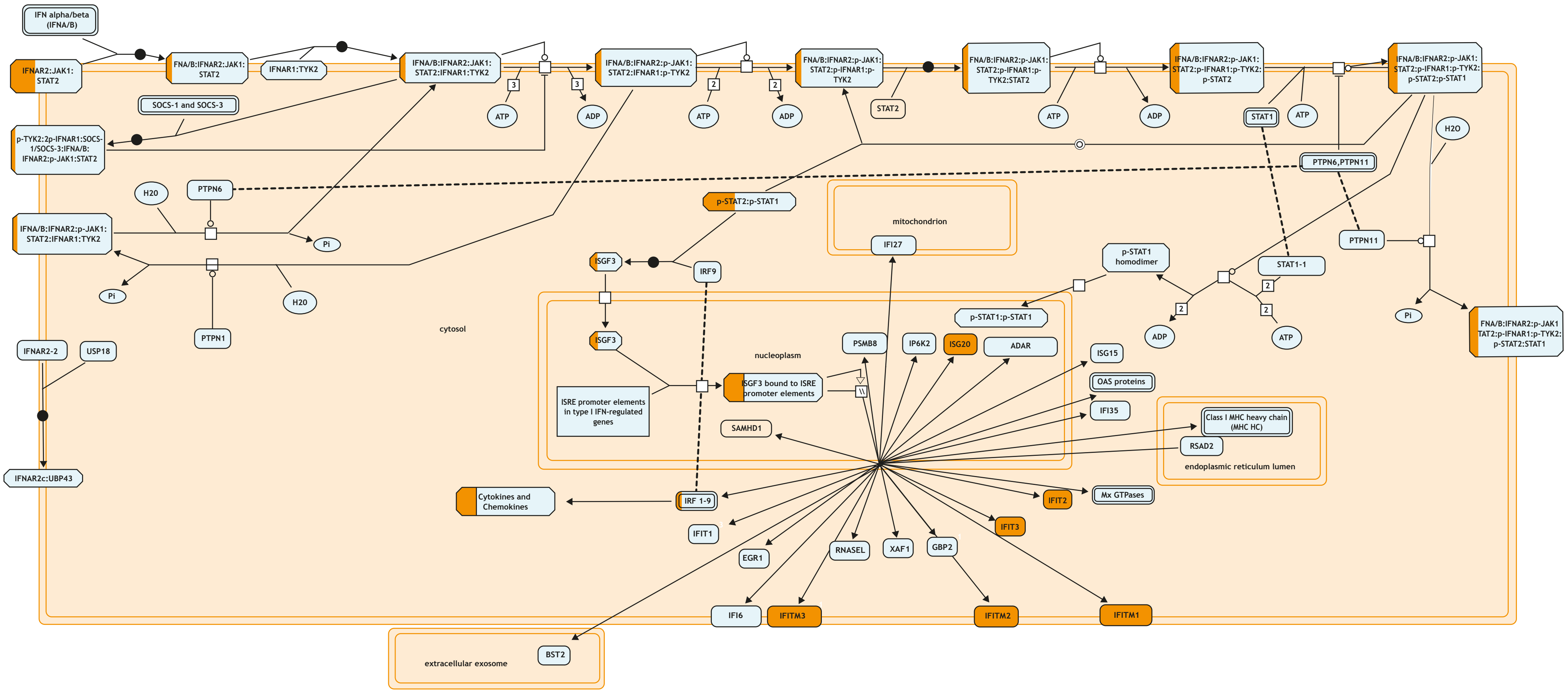

Figure S6

Representation of "Type I IFN" pathway. In orange are represented all the DE transcripts at d7. Boxes containing more genes have a partial orange coloration depending on the content of DE transcripts.
